# OpenSim Moco: Musculoskeletal optimal control

**DOI:** 10.1371/journal.pcbi.1008493

**Published:** 2020-12-28

**Authors:** Christopher L. Dembia, Nicholas A. Bianco, Antoine Falisse, Jennifer L. Hicks, Scott L. Delp

**Affiliations:** 1 Department of Mechanical Engineering, Stanford University, Stanford, California, United States of America; 2 Department of Movement Sciences, KU Leuven, Leuven, Belgium; 3 Department of Bioengineering, Stanford University, Stanford, California, United States of America; 4 Department of Orthopaedic Surgery, Stanford University, Stanford, California, United States of America; University of Kentucky, UNITED STATES

## Abstract

Musculoskeletal simulations are used in many different applications, ranging from the design of wearable robots that interact with humans to the analysis of patients with impaired movement. Here, we introduce OpenSim Moco, a software toolkit for optimizing the motion and control of musculoskeletal models built in the OpenSim modeling and simulation package. OpenSim Moco uses the direct collocation method, which is often faster and can handle more diverse problems than other methods for musculoskeletal simulation. Moco frees researchers from implementing direct collocation themselves—which typically requires extensive technical expertise—and allows them to focus on their scientific questions. The software can handle a wide range of problems that interest biomechanists, including motion tracking, motion prediction, parameter optimization, model fitting, electromyography-driven simulation, and device design. Moco is the first musculoskeletal direct collocation tool to handle kinematic constraints, which enable modeling of kinematic loops (e.g., cycling models) and complex anatomy (e.g., patellar motion). To show the abilities of Moco, we first solved for muscle activity that produced an observed walking motion while minimizing squared muscle excitations and knee joint loading. Next, we predicted how muscle weakness may cause deviations from a normal walking motion. Lastly, we predicted a squat-to-stand motion and optimized the stiffness of an assistive device placed at the knee. We designed Moco to be easy to use, customizable, and extensible, thereby accelerating the use of simulations to understand the movement of humans and other animals.

## Author summary

Computer simulation has become an increasingly popular tool for studying the musculoskeletal system. Simulations are used to study the role of muscles in walking and running, to analyze the gait of individuals with neurological disease, and to design prostheses and exoskeletons. Historically, researchers have relied on experimental data to generate simulations and estimate muscle activity. Modern simulation approaches based on the direct collocation optimal control method allow researchers to not only estimate muscle activity, but also predict new motions without the need for experimental data. However, direct collocation methods are difficult and time-consuming to implement and require expertise in optimal control and optimization theory. Here we introduce OpenSim Moco, an open source software package that makes predicting new motions accessible to those without an optimal control background. Moco leverages the existing modeling tools offered by the OpenSim musculoskeletal modeling package and provides an easy-to-use interface that facilitates generating and sharing simulation pipelines. Moco is modular and easily extensible and includes a testing suite that solves problems with known solutions. We provide examples including predicting muscle activity that minimizes knee loading, predicting how muscle weakness affects normal walking, and optimizing a knee exoskeleton to assist a squat-to-stand motion.

This is a *PLOS Computational Biology* Software paper.

## Introduction

Musculoskeletal simulations have shed light on movement disorders by, for example, discovering ways to walk that reduce knee loading [1], revealing that children with cerebral palsy exhibit simplified motor control when walking [2], and reproducing eye disorders that cause diplopia [3]. Beyond human health and performance, researchers use musculoskeletal simulations to understand how nonhuman animals move, for example by studying dinosaur locomotion [4] and differences between human and chimpanzee strength [5].

Simulations of movement are often categorized by whether the motion is prescribed from experimental data or predicted by the simulation. One may prescribe the motion of a musculoskeletal model to match a motion measured in an experiment [6, 7] to estimate unmeasured quantities such as muscle-level energy consumption [8, 9]. The most commonly used prescribed motion methods are rapid but lack support for important model features such as muscle dynamics (e.g., static optimization [10]). Motion prediction [11] can establish cause-effect relationships (e.g., discovering gait impairments that arise from weakness or contracture [12]) and help design clinical interventions (e.g., prostheses [13] and exoskeletons [14]). However, some methods for predicting motions, such as single shooting, often require waiting many hours or days for a solution, so researchers often simplify the musculature of their model or reduce the dimensionality of the control scheme [12, 15]. A third category, which lies between prescribing and predicting motion, is tracking motion, where errors between model kinematics and reference data are part of the cost function rather than exactly prescribed [16]. While some researchers have created their own code to solve problems spanning the prescribed-to-predicted spectrum, many researchers lack the expertise or resources required to implement custom algorithms. Simulation methods that solve diverse problems, combined with tools that make these methods easy to adopt for all researchers, would accelerate the application of simulations to scientific and clinical questions.

Most musculoskeletal simulations are naturally posed as optimal control problems [17]. Optimal control problems seek the parameters and time-varying controls of a system that minimize a cost (e.g., energy consumption) subject to the system dynamics, expressed as differential-algebraic equations. Biomechanists often use single shooting to solve optimal control problems, but more rapid alternatives exist. Direct multiple shooting uses time-stepping numerical integration to simulate consecutive intervals of a trajectory and constrains adjacent interval endpoints to be consistent [18, 19], which leads to improved numerical stability and faster convergence compared to single shooting. Direct collocation is an increasingly popular method that avoids the need for time-stepping integration and permits a more easily configurable trade-off between accuracy and computational cost compared to direct multiple shooting. With this method, the states and controls of the system are approximated as polynomial splines over a mesh of time points and an optimizer solves for the knot points that lead the splines to obey the system dynamics [20–23]. The dynamics are enforced by requiring the time derivative of the state splines to match the derivative from the system differential equations at specified time points. (This method is called “direct collocation” because the spline derivatives are “collocated” with the exact derivatives ([24], page 211; [25], page 498).) Direct collocation produces a nonlinear program in which the states are introduced as variables and the system dynamics are enforced as constraints. Typical musculoskeletal models lead to optimization problems with thousands of variables. These large problems are tractable with direct collocation because the constraints enforcing the system dynamics at a given time depend only on the variables near that time.

The advantages of direct collocation have led biomechanists to use the method for prescribing motions [26, 27], tracking motions [16, 28–31], predicting motions [32–44], fitting muscle properties [45], and optimizing model parameters [46]. Researchers have made key methodological advances, including efficiently handling multibody and muscle dynamics via implicit formulations [26, 47], minimizing energy consumption [48, 49], and employing algorithmic differentiation to simulate complex models more rapidly compared to using finite differences [50].

Despite the advantages of direct collocation, very few biomechanics laboratories have been able to apply this powerful technique. The method requires arduous bookkeeping of variables and efficient calculation of the objective and constraint function derivatives required by gradient-based optimization algorithms. Several direct collocation solvers exist (e.g., [51, 52]), but current solvers require users to incorporate their musculoskeletal models manually. OpenSim is a software package used by thousands of biomechanics researchers to model musculoskeletal systems [53–55], but OpenSim does not currently employ direct collocation. Several biomechanists have graciously shared their code combining OpenSim or hand-coded models with direct collocation solvers, but such code is tailored to specific models or problem formulations, or lacks support for models with kinematic constraints [26, 35, 36, 40]. Furthermore, such code can depend on closed-source components, which can limit custom extensions [26]. Lastly, choosing the problem formulation (e.g., expressing dynamics as explicit or implicit differential equations) and solver settings (e.g., detecting sparsity patterns automatically) that lead to fast convergence requires expertise; ideally, such expertise is embedded into the software via defaults, and users can edit their formulation or solver settings with simple commands.

**Fig 1:**
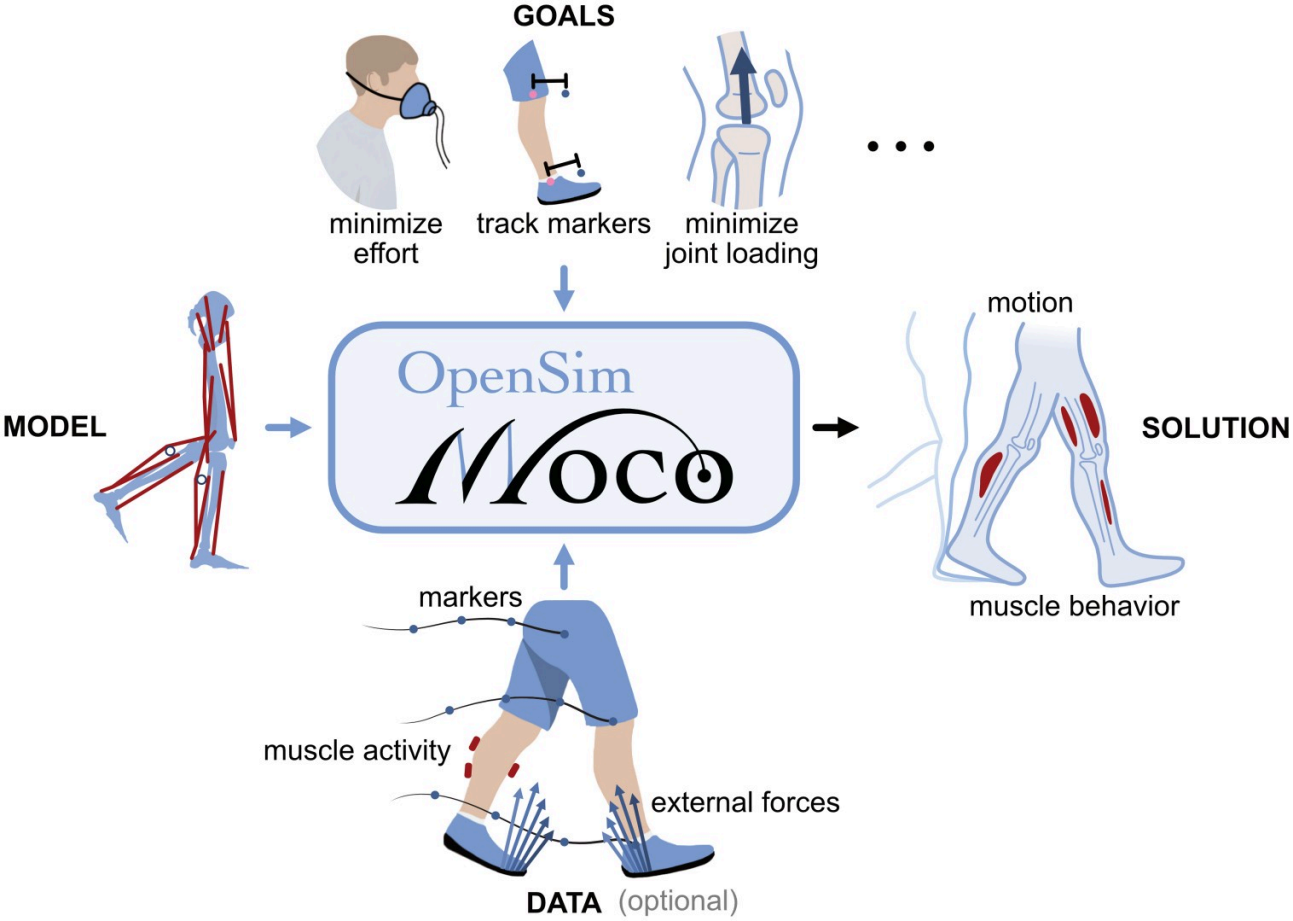
Overview of Moco. OpenSim Moco produces the optimal motion and muscle behavior for an OpenSim musculoskeletal model [54], given goals to achieve during the motion and reference data. Moco provides a library of goals, such as minimizing effort (illustrated with an indirect calorimetry mask), deviation from marker data (or generalized coordinate data), and joint loading. Reference data for the motion (markers or generalized coordinates), external forces (from force places), and muscle activity (from electromyography) are optional. Illustration credit: Kai Rasmussen.

To improve the accessibility of advanced optimal control methods in musculoskeletal biomechanics, we introduce OpenSim Moco (“musculoskeletal optimal control”), an easy-to-use, customizable, and extensible software toolkit for solving optimal control problems with OpenSim musculoskeletal models (Fig 1). OpenSim frees biomechanists from implementing equations of motion on their own, and Moco frees biomechanists from implementing direct collocation. Moco not only removes the need to set up gradient-based optimization, but also provides an interface that abstracts away the details of constructing an optimal control problem. Users can add custom cost terms or constraints if Moco does not provide what they need. This paper details the design and implementation of Moco and shows three applications. First, we estimated muscle activity for an observed walking motion and predicted changes in muscle activity with a different cost function. Second, we used Moco to predict how muscle weakness may cause deviations from a normal walking motion. Finally, to illustrate that Moco can predict motions and model parameters, we predicted a squat-to-stand motion and optimized the stiffness of a passive assistive device.

## Design and implementation

**Fig 2:**
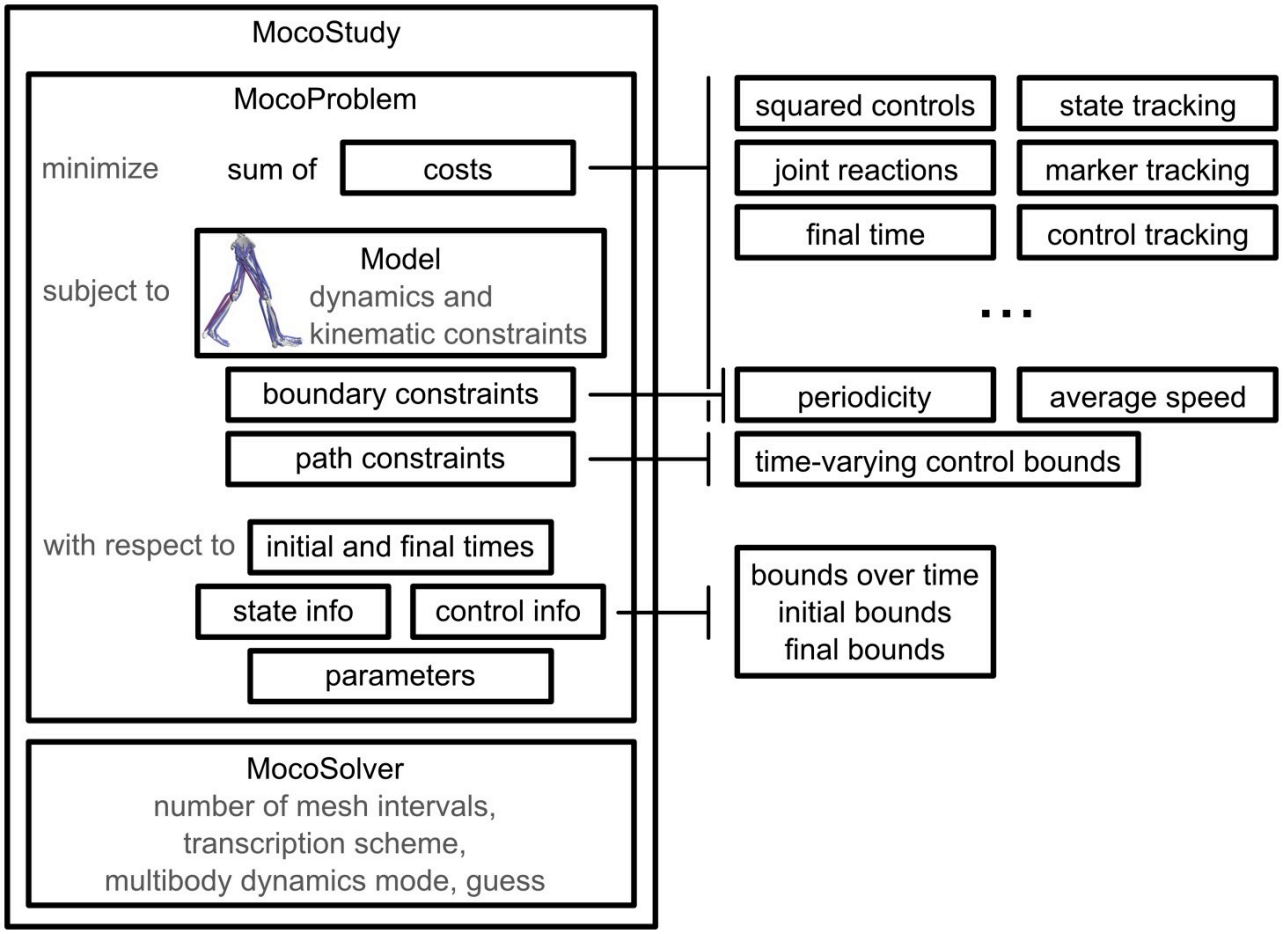
Overview of *MocoStudy*. Researchers can use Moco to solve custom optimal control problems via a library of cost, boundary constraint, and path constraint modules. Moco contains additional cost modules beyond what is shown here, and users can define their own custom modules.

Researchers can use Moco to solve optimal control problems that they define via a library of cost and constraint modules, which are implemented through configurable software classes. Users describe their problem with the *MocoProblem* class. (We use italics to denote the names of classes in Moco and OpenSim.) To decouple the problem from the numerical methods used to solve it, we use the *MocoSolver* class. Moco classes are available via C++, MATLAB, Python, and XML text files, with interfaces familiar to OpenSim users. We package the *MocoProblem* and *MocoSolver* together into a *MocoStudy* (Fig 2), which can be written to and loaded from XML text files. Moco contains utilities for visualizing and plotting the solution to a study, which is held by the *MocoSolution* class. For certain standard biomechanics problems, Moco provides simpler interfaces that may be preferable to the flexibility of *MocoStudy*.

### Defining problems with *MocoProblem*

*MocoProblem* supports a diversity of scientific questions and contains the following elements.

- **cost terms**: Users can minimize a weighted sum of squared controls, deviation from an observed motion, joint reaction loads, the duration of a motion, and other costs by appending to the *MocoProblem* an instance of the class associated with a cost module (e.g., *MocoControlGoal* allows minimizing the sum of squared controls).
- **multibody dynamics, muscle dynamics, and kinematic constraints**: OpenSim *Models* are a popular format for modeling musculoskeletal systems. Moco uses OpenSim *Models* to obtain the underlying differential-algebraic system of equations, including multibody dynamics, auxiliary dynamics (e.g., muscle activation dynamics and tendon compliance), and kinematic constraints; these equations are provided by the industrial-grade Simbody multibody dynamics library [55]. Moco supports any kinematic constraints present in the system using a method by Posa and colleagues [56]. Kinematic constraints are useful for modeling anatomy that either is not easily described using standard joints or would otherwise require calibrated models of ligaments and cartilage to produce the salient features of the desired motion. Examples of models with complex anatomy achieved by kinematic constraints include models of the knee, shoulder, and neck [57–60]. Kinematic constraints are also necessary when modeling closed kinematic loops. For example, cycling models can create closed kinematic loops if both feet are fixed to the same bicycle crank [44].
- **boundary constraints**: Users can enforce average speed, symmetry, or periodicity with constraints relating initial and final states.
- **path constraints**: Users can constrain any function of time to lie in a specified range over the motion. For example, researchers often estimate muscle activity with electromyography and Moco allows constraining simulated muscle excitations to be close to those measurements via the *MocoControlBoundConstraint* class.
- **parameter optimization**: Users can optimize model properties, such as the mass of a body, the optimal fiber length of a muscle, or the stiffness of an exoskeleton.
- **bounds on variables**: Users can bound the values of states, controls, and initial and final time.

For a detailed mathematical description of the problems supported by Moco, including how Moco handles kinematic constraints, consult S1 Appendix.

Users can combine the modules of a *MocoProblem* in diverse ways. The following examples illustrate the breadth of problems that users can solve.

- **dynamically-constrained inverse kinematics**: Minimize the error between experimental and model marker positions (marker tracking) and squared controls while obeying multibody dynamics.
- **electromyography-constrained muscle force estimation**: For a prescribed experimental motion, minimize squared muscle excitations while obeying multibody dynamics and constraining the difference between muscle excitations and electromyography data.
- **torso mass calibration**: For a prescribed experimental motion, adjust the mass of the torso to minimize residual forces at the pelvis while obeying multibody dynamics.
- **prediction of kinematic and muscular coordination adaptations to walking in an exoskeleton**: Minimize squared muscle excitations while obeying multibody and muscle dynamics and a constraint on average speed of the mass center.

**Fig 3:**
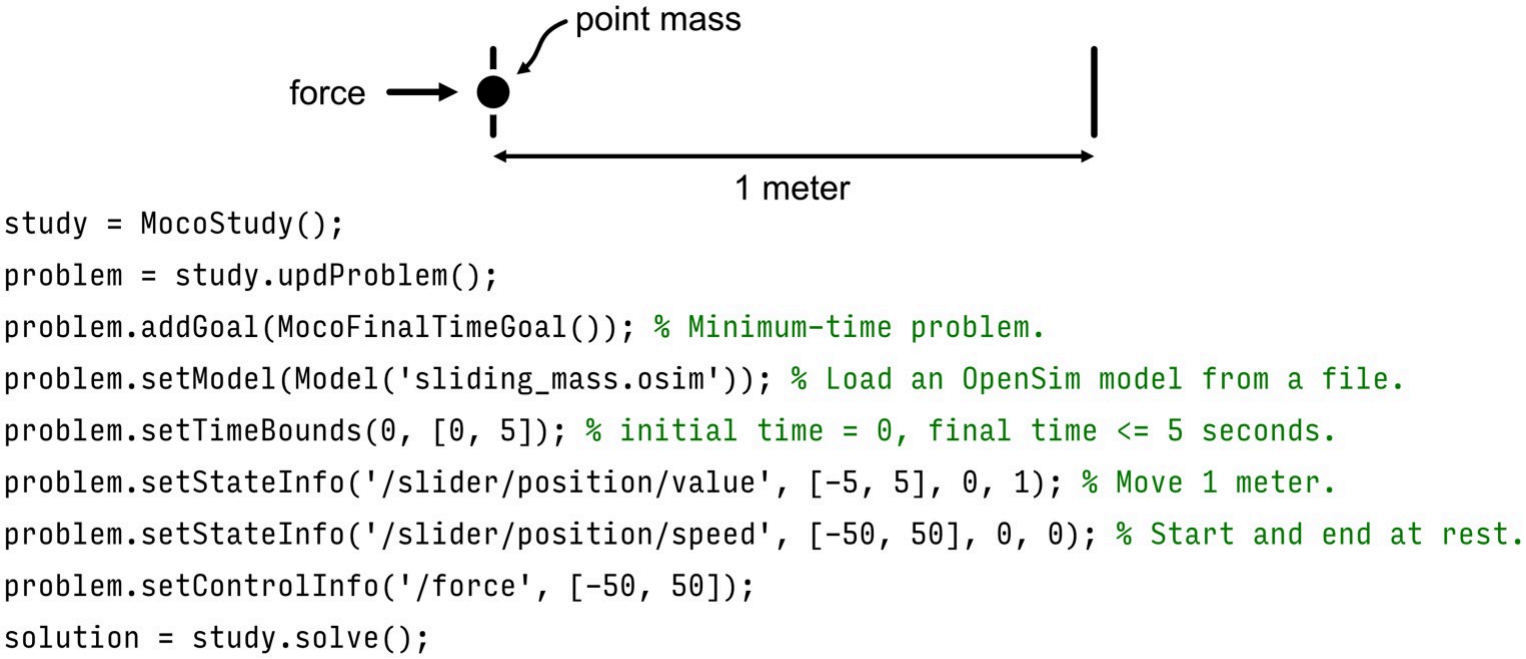
Example code for a simple problem. This MATLAB code uses Moco to find the force to apply to a point mass to move the mass by one meter (starting and ending at rest) in minimum time. Solving this problem requires only 9 lines of code.

We demonstrate the Moco interface in Fig 3 with a simple MATLAB example. Setting the model and adding a cost (termed “goal” in the code) each require only a single statement.

Direct collocation relies on gradient-based optimization, which converges faster and more reliably when all functions in the problem are continuous and differentiable. Moco includes a previously published continuous and differentiable muscle model named *DeGrooteFregly2016Muscle* [26]. We extended this muscle model to include damping, which further improves convergence but can introduce small compressive forces when the muscle fiber shortens at low activation. To represent compliant contact forces in a continuous and smooth manner, Moco includes a previously published contact model named *SmoothSphereHalfSpaceForce* [61].

Users wishing to employ a cost term, boundary constraint, or path constraint that Moco does not provide can create a C++ plugin using the same steps as for OpenSim plugins. By providing a library of cost, boundary constraint, and path constraint modules, allowing these modules to be combined, and enabling users to create their own modules, we achieve our design goals of ease-of-use, customizability, and extensibility.

### Solving problems with *MocoSolver*

All details of solving an optimal control problem are encapsulated in *MocoSolver*, which is decoupled from *MocoProblem* for flexibility. For example, users can add bodies or muscles to their model without modifying the solver. *MocoSolver* uses the CasADi library [62] to transcribe the continuous optimal control problem defined by *MocoProblem* into a finite-dimensional nonlinear program, which we solve with well-established gradient-based nonlinear program solvers such as IPOPT [63] and SNOPT [64] (see S1 Appendix). CasADi can provide the derivatives of the objective and constraint functions using either finite differences or algorithmic differentiation; Moco uses only finite differences to avoid the complexity of adapting the OpenSim codebase to support algorithmic differentiation.

Moco provides two transcription schemes: the second-order trapezoidal scheme and the third-order Hermite–Simpson scheme [20]. Multibody dynamics can be expressed with either explicit differential equations (“forward dynamics”) or implicit differential equations (“inverse dynamics”); problems may converge faster with implicit differential equations [26, 47]. *MocoSolver* also provides settings for the constraint tolerance, convergence tolerance, and the number of mesh intervals used to solve the *MocoProblem*.

**Fig 4:**
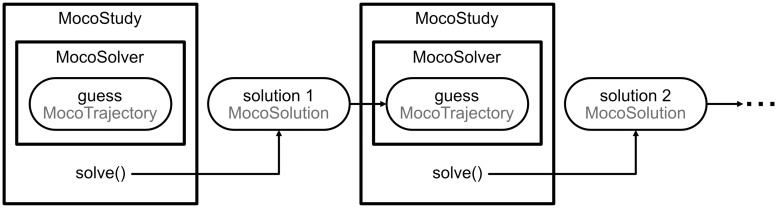
Using trajectories to solve problems iteratively. Guesses for the optimization are specified using *MocoTrajectory*, which holds the values of states, controls, and parameters at any iteration in the optimization. *MocoSolution* is a subclass of *MocoTrajectory* that holds the solution to a study and includes the success status of the optimization, the final objective value, and the number of solver iterations. Users can use the solution of one problem as the initial guess for a subsequent problem.

Solving a *MocoStudy* yields a *MocoSolution* (Fig 4), which is a subclass of *MocoTrajectory* and provides easy access to the values of all variables at any iteration in the optimization. Users provide initial guesses via *MocoTrajectory*, and can use the solution from one problem as the initial guess for a subsequent problem; this allows users to build a complex study with a series of simpler studies. For example, predicting the change in walking kinematics caused by an ankle exoskeleton could benefit from an initial guess generated from a tracking simulation of normal walking. *MocoSolution* provides additional information, including whether or not the solver converged, the final objective value, and the number of solver iterations.

After solving a problem, researchers can use Moco to visualize the solution as an animation, plot the state and control trajectories (with utilities provided in MATLAB and Python), or compute quantities from the solution. The ability to save *MocoTrajectories* and *MocoStudies* to files allows users to reproduce each other’s results, which is essential for sound science [65].

### Tools for standard problems

**Fig 5:**
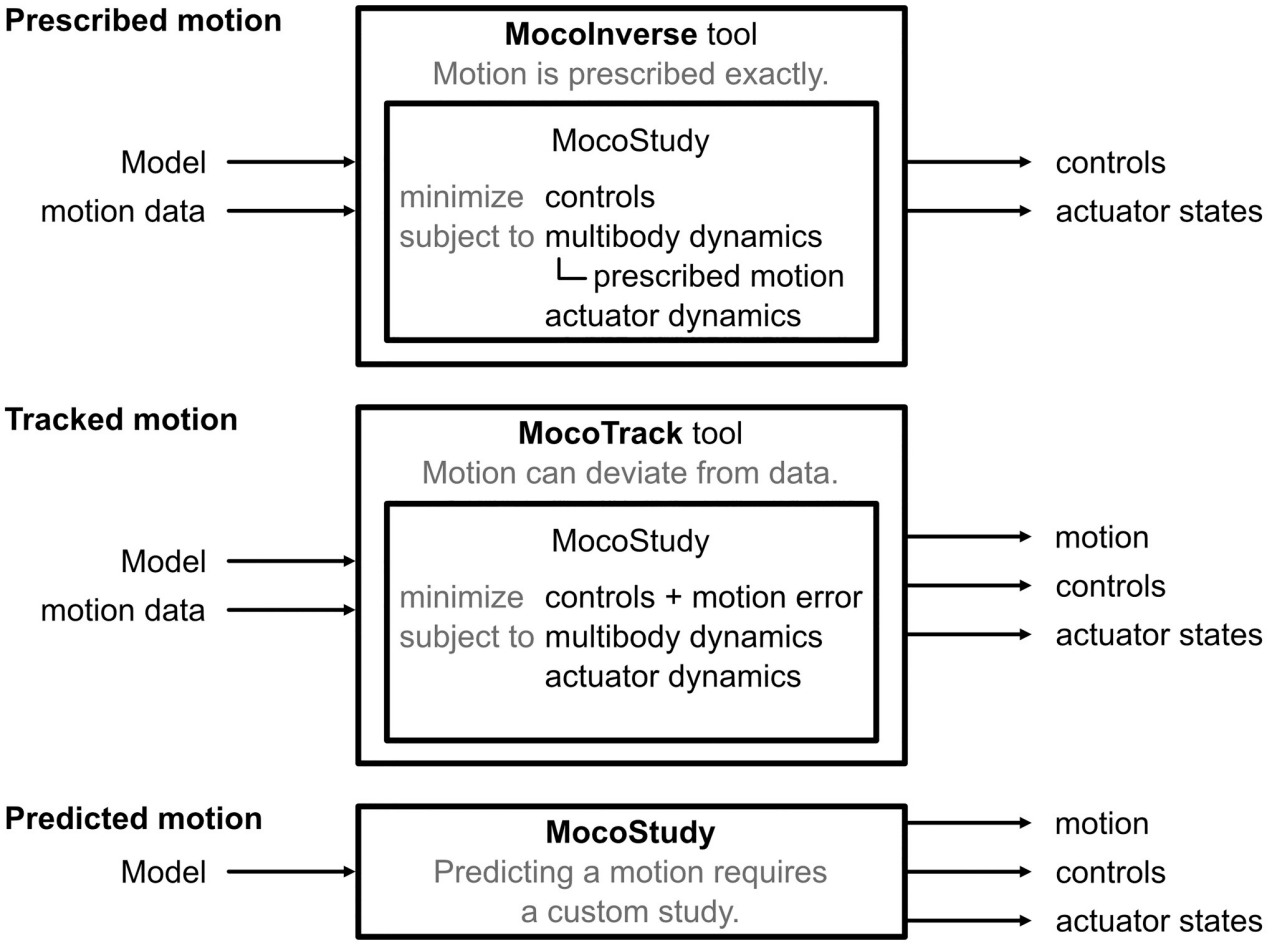
Solving prescribed motion, tracked motion, and predicted motion problems. Moco provides the tools *MocoTrack* and *MocoInverse* for solving standard problems. Both require a *Model* and kinematic data as inputs and produce controls and actuator states as outputs, but these tools solve different optimal control problems. *MocoTrack* produces a new simulated motion, while *MocoInverse* does not permit deviations from the provided kinematic data. Predicting a motion is not easily standardized and requires a custom *MocoStudy*.

Currently, Moco provides two tools for solving standard problems (Fig 5):

- *MocoInverse* solves the muscle/actuator redundancy problem, wherein we solve for muscle (or other actuator) controls that achieve a motion that is prescribed exactly (see S1 Appendix) while minimizing squared controls or other costs.
- *MocoTrack* solves motion tracking problems, wherein we solve for both a motion and muscle (or other actuator) controls that minimize the error compared to an observed motion in addition to squared controls or other costs.

*MocoTrack* is useful for predicting deviations from motion data (e.g., predicting kinematic adaptations to an exoskeleton), while *MocoInverse* is a faster option when the motion should be enforced exactly (e.g., estimating elastic energy storage for an observed motion). *MocoTrack* can use contact models, while *MocoInverse* can apply measured external forces to the model. For both tools, the only required inputs are an OpenSim model and motion data (coordinate or marker trajectories, and external forces if relevant). Internally, the tools build a *MocoStudy* with solver settings that yield fast and reliable convergence on problems we tested.

Future versions of Moco may include tools for model calibration, electromyography-driven simulation, and other standard problems. For problems that do not fit into a standard form, such as predicting a motion, *MocoStudy* provides the necessary flexibility.

### Verification

Verifying software is essential [66]; thus, we conducted extensive verification tests. For example, to verify that Moco implements direct collocation correctly we solved the “linear tangent steering” optimal control problem [67], which has a known solution. The solution matched the known solution with a root-mean-square error of 2.7 × 10^−5^.

**Fig 6:**
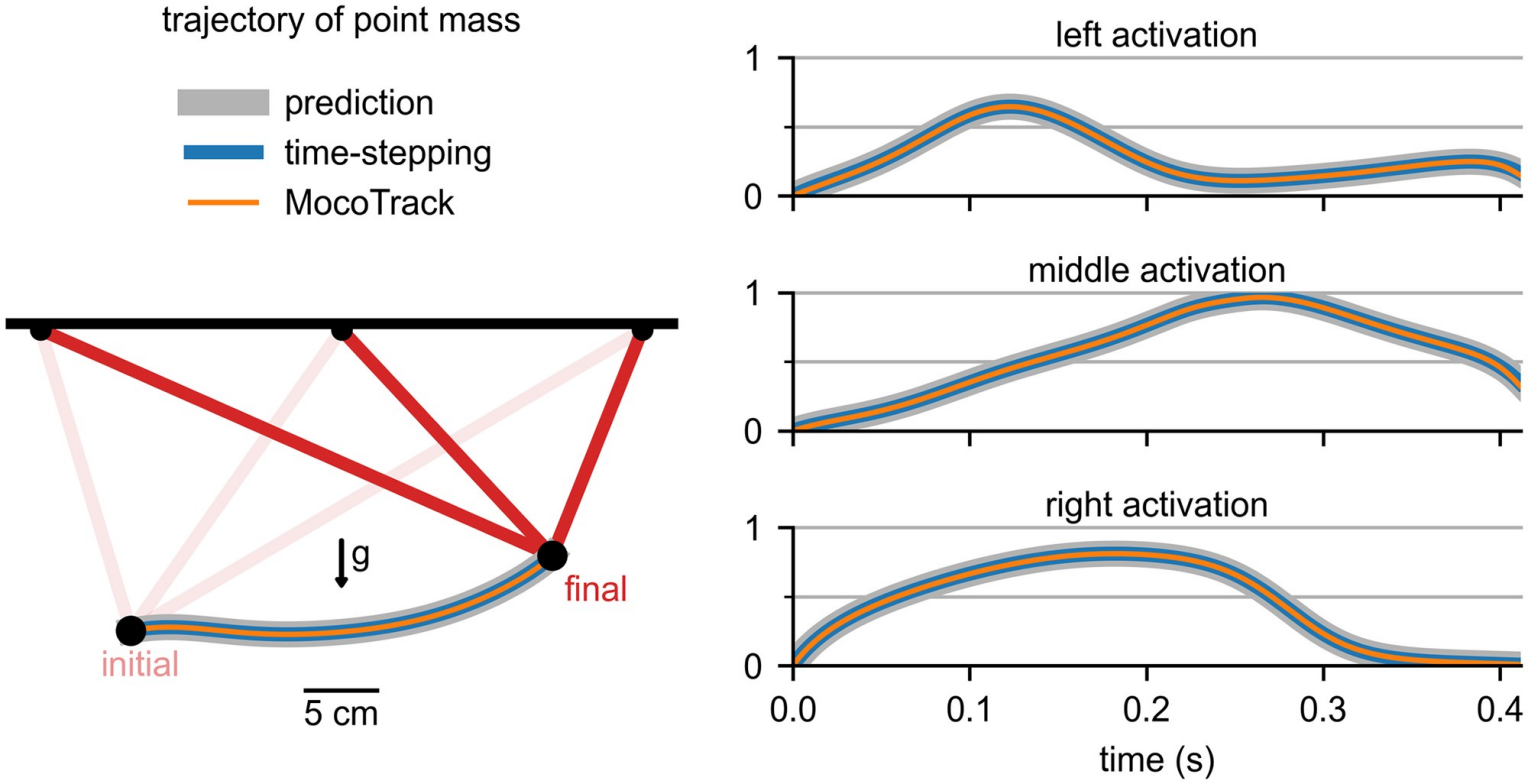
Verification of time-stepping and motion tracking. Left: The trajectory of a point mass suspended by three muscles and moving under the influence of gravity (*g*) was simulated using Moco. Right: The activations of the “left,” “middle,” and “right” muscles are shown for different simulations. The original trajectory (gray band) was predicted by minimizing final time and the sum of squared muscle excitations. The time-stepping forward simulation driven with the predicted controls (blue) produced the motion we originally predicted. Tracking the predicted motion with *MocoTrack* (orange) produced the original activations.

We also ensured that a time-stepping forward simulation using controls from a motion prediction produced the same motion as in the prediction. This test used a model consisting of a point mass suspended by three muscles (*DeGrooteFregly2016Muscle*) and under the influence of gravity (Fig 6). For this problem, the muscles had activation dynamics and rigid tendons. We first predicted the state and control trajectories to move the point mass between prescribed initial and final positions, starting and ending at rest (Fig 6, gray band). In this prediction, the cost included both the sum of squared muscle excitations and the final time. Then, we used the predicted controls to perform a time-stepping forward simulation using an OpenSim integrator (Fig 6, blue line). The resulting position trajectory of the point mass matched that from the prediction with a root-mean-square error of 0.0051 m (1.8% of the distance between the initial and final positions). This gives us confidence that Moco enforces the same multibody and muscle dynamics enforced in a time-stepping forward simulation in OpenSim. For more complex problems, conducting a time-stepping forward simulation using controls from a *MocoSolution* requires a stabilizing feedback controller to counteract numerical errors.

To gain confidence in motion tracking problems, we ensured that using *MocoTrack* on a synthesized motion with known muscle activity produced the original muscle activity. We used the same suspended point mass model and tracked the previous motion prediction (Fig 6, gray band) while minimizing squared excitations. The muscle activations from *MocoTrack* (Fig 6, orange line) matched those from the prediction with a root-mean-square error that was 0.47% of the peak predicted activation. For most tracking problems of interest, we do not know the true muscle activity solution; verifying tracking problems using synthesized data gives us confidence in the ability of Moco to estimate muscle activity.

Moco contains an automated test suite, with over 80 test cases, that extends beyond the verification described here. The test suite contains four major types of tests: analytical tests, interface tests, algorithmic tests, and regression tests. All tests are located within the source code; we employ continuous integration to ensure all tests succeed before accepting changes to the code on GitHub. A detailed description of our automated test suite is in S1 Appendix.

Our automated test suite can give users confidence that Moco implements direct collocation correctly, but users must ensure that they use appropriate mesh density (number of mesh intervals per unit time) and constraint tolerance settings for their problems [66]. While a problem with a coarse mesh may meet the user’s specified constraint tolerance, errors in the dynamics may be too large for the simulation to be useful ([20], section 4.7). Users can perform convergence analyses to ensure that solutions are not overly sensitive to the chosen mesh density or constraint tolerance.

## Results

Here we present three results obtained using Moco that represent biomechanics applications spanning the prescribed-to-predicted spectrum. In the first result, we used the *MocoInverse* tool to solve for muscle activity while prescribing exactly the model generalized coordinate values. We compared muscle activity results using cost functions with and without a knee contact loading term. In the second result, we used *MocoTrack* to simulate normal walking and walking with weakened hip abductor and ankle dorsiflexor muscles. When simulating weakness, we weakened muscles (while retaining the original cost function weights) until the model was no longer able to track the normal kinematic reference data and compared the resulting motion to known gait pathologies. In the third result, we predicted a squat-to-stand motion using a custom *MocoStudy*. We then solved the problem again while optimizing the stiffness of an assistive device at the knee to evaluate the effect of assistance on muscle activity. These three results illustrate the most common user interfaces provided by Moco and demonstrate that Moco can efficiently and accurately solve problems relevant to biomechanics researchers.

### Prescribing a walking motion to estimate muscle activity

**Fig 7:**
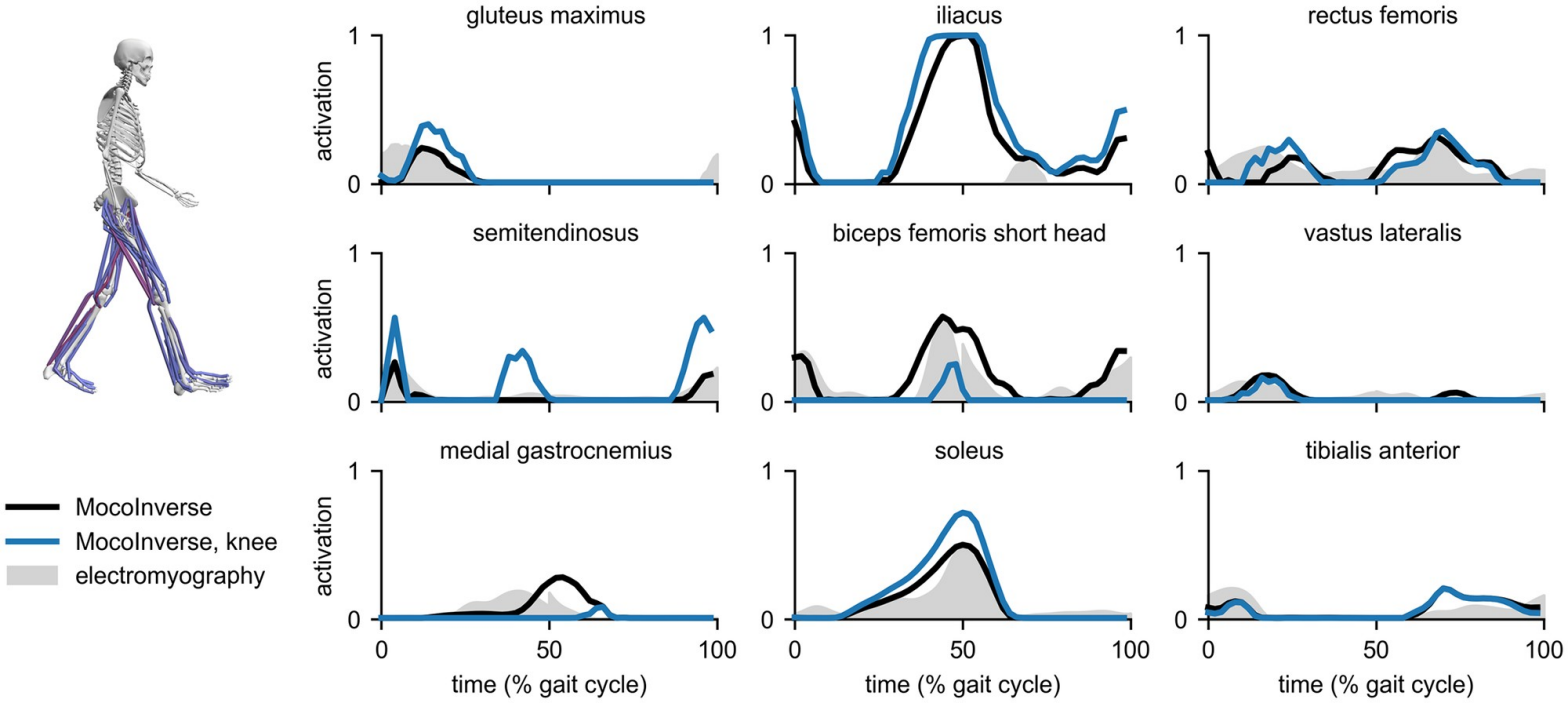
Estimates of muscle activity during walking. *MocoInverse* produced muscle activations (black) whose timing was similar to the timing from electromyography measurements (gray) for all muscles shown except iliacus. Electromyography for gluteus maximus and iliacus comes from Perry and Burnfield [68]; electromyography for all other muscles comes from Rajagopal et al. [59] and was normalized such that its peak matched the peak of the *MocoInverse* activations. Adding a cost term for knee joint loading reduced the activity of the biceps femoris short head and medial gastrocnemius, which span the knee (blue), and increased the activity of other muscles to ensure the original prescribed motion was achieved.

Moco can estimate muscle activity in walking, which allows studying muscle coordination in normal and pathological gait. We used a model with 29 degrees of freedom and 80 lower-limb muscles, generalized coordinate trajectories (obtained from an inverse kinematics solution and adjusted using the OpenSim Residual Reduction Algorithm), and ground reaction forces to simulate one gait cycle of walking at a self-selected speed of 1.24 m/s. The model and data are based on [59], where they are described in greater detail. We modeled activation dynamics in all muscles but only modeled tendon compliance in the gastrocnemii and soleus, where a rigid-tendon assumption is less appropriate [26]. We solved for muscle behavior using *MocoInverse*, which prescribes kinematics exactly (see “Tools for standard problems” for details). The initial guess for all variables was the midpoint of the bounds on the variables. We compared the muscle activations produced by *MocoInverse* to electromyography measurements (Fig 7; black lines and gray shading).

*MocoInverse* produced activations that included some of the major features of the electromyography data, such as the timing of peak activity for the semitendinosus, biceps femoris short head, vastus lateralis, medial gastrocnemius, and soleus. Activity for the iliacus was much higher than measured in a separate data set [68]; this is likely because the passive hip flexion moment generated by the model during late stance [59] is much lower than expected based on passive moments measured by load cells [69]. With *MocoInverse*, the difference between required net joint moments and muscle-generated net joint moments (termed “reserve” moments) were restricted to be no greater than 2.5 N-m across time and degrees of freedom, which is in accordance with established guidelines (reserve moments are below 5% of peak net joint moments [66]). *MocoInverse* solved this problem in 3.5 minutes using a 3.6 GHz Intel Core i7 processor with 8 parallel threads. We compared the solution from *MocoInverse* to those from the OpenSim Static Optimization and Computed Muscle Control [6] tools (S1 Fig). Muscle activity was similar between all three tools for all muscles shown except the medial gastrocnemius, soleus, and tibialis anterior; differences for these muscles were caused by differences in the algorithms, such as how tendon compliance was handled.

To demonstrate that Moco problems are customizable, we added a cost term to *MocoInverse* to minimize knee joint loading (weight for squared controls term: 1.0; weight for joint loading term: 0.005). With this additional cost, the average magnitude of the knee joint reaction force decreased from 1.7 to 1.1 body weights. The peak knee joint reaction force was 5.0 body weights with and without the additional cost; obtaining a peak value closer to those measured with in-vivo knee joint implants (1.8–3.0 body weights [70]) may be possible by minimizing the deviation of muscle activity from electromyography data [71]. As expected, the activity of muscles crossing the knee joint (vastus lateralis, biceps femoris short head, and medial gastrocnemius) decreased (Fig 7; blue lines). To compensate for the reduced medial gastrocnemius moment at the ankle, soleus activity increased, as seen in a previous simulation study [72]. Moco solved this problem in 15 minutes.

### Tracking a walking motion with normal and weakened muscles

In addition to producing initial guesses for predictive optimizations, tracking problems (in which a motion is tracked as part of the cost function rather than exactly prescribed) can be useful for studying how changes to a model affect its ability to reproduce a reference motion. Moco contains the *MocoTrack* tool for constructing and solving tracking simulations. We explored the effect of weakening different muscle groups in a lower extremity walking model on the ability to track normal walking data. As inputs to *MocoTrack*, we used the same model and kinematic data as for the *MocoInverse* problem above. Instead of prescribing ground reaction forces, we added compliant foot–ground contact force elements on both feet [43]. We removed the torque actuators serving as “reserve” moments, which were only necessary for *MocoInverse*, so that the lower limbs were entirely muscle-driven. Unlike *MocoInverse*, *MocoTrack* does not prescribe model generalized coordinate values; therefore, we enforced the kinematic constraints in the model that dictate patellar motion based on the knee angle [73]. These kinematic constraints enabled realistic muscle moment arms about the knee without the need to model knee ligaments and cartilage. The cost included terms for motion tracking (weight: 0.11) and squared controls (weight: 0.16). We included a boundary constraint that ensured the model walked with the same average speed as in the reference data, and a path constraint that prevented the feet from interpenetrating. We assumed mediolateral symmetry and simulated half of a gait cycle. The initial guess for the kinematic variables was taken from the tracking data, and the initial guess for muscle-related variables was the midpoint of the bounds on the variables.

**Fig 8:**
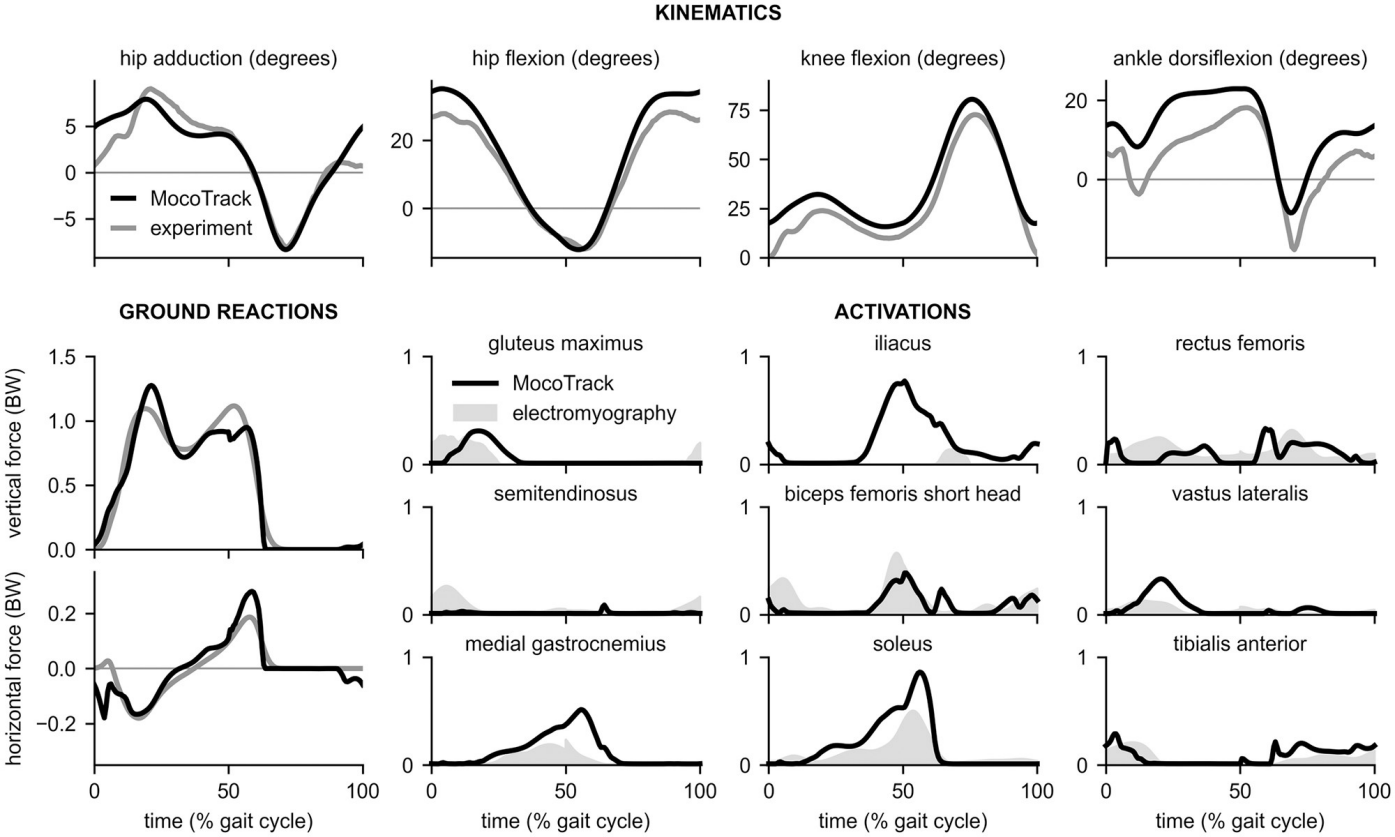
Tracking 3-D experimental motion data. Top: *MocoTrack* produced kinematics (black) that tracked experimental data (gray). Left: *MocoTrack* produced sagittal plane ground reaction forces (black) whose timing matched that of experimentally-measured ground reaction forces (gray). However, the magnitude in the first peak of the vertical ground reaction force was overestimated. Right: Similar to *MocoInverse*, *MocoTrack* produced muscle activations (black) that matched the timing of electromyography measurements (gray). Electromyography for gluteus maximus and iliacus comes from Perry and Burnfield [68]; electromyography for all other muscles come from Rajagopal et al. [59] and signals were again normalized such that their peak matched the peak of the *MocoInverse* activations to be consistent with Fig 7.

We first solved the tracking problem using normal muscle strengths (maximum isometric forces). *MocoTrack* produced a motion that tracked experimental kinematics (Fig 8, top) and ground reaction forces whose timing matched that of the sagittal plane experimental ground reaction forces (Fig 8, left). Muscle activations for many major lower extremity muscles were qualitatively similar to electromyography data including gluteus maximus, biceps femoris short head, vastus lateralis, medial gastrocnemius, soleus, and tibialis anterior (Fig 8, activations). Using the same computer as for the *MocoInverse* problems, *MocoTrack* solved this problem in 130 minutes; this duration is shorter than that commonly seen in single shooting predictions [12], but is longer than what can be achieved when combining algorithmic differentiation with direct collocation [43].

**Fig 9:**
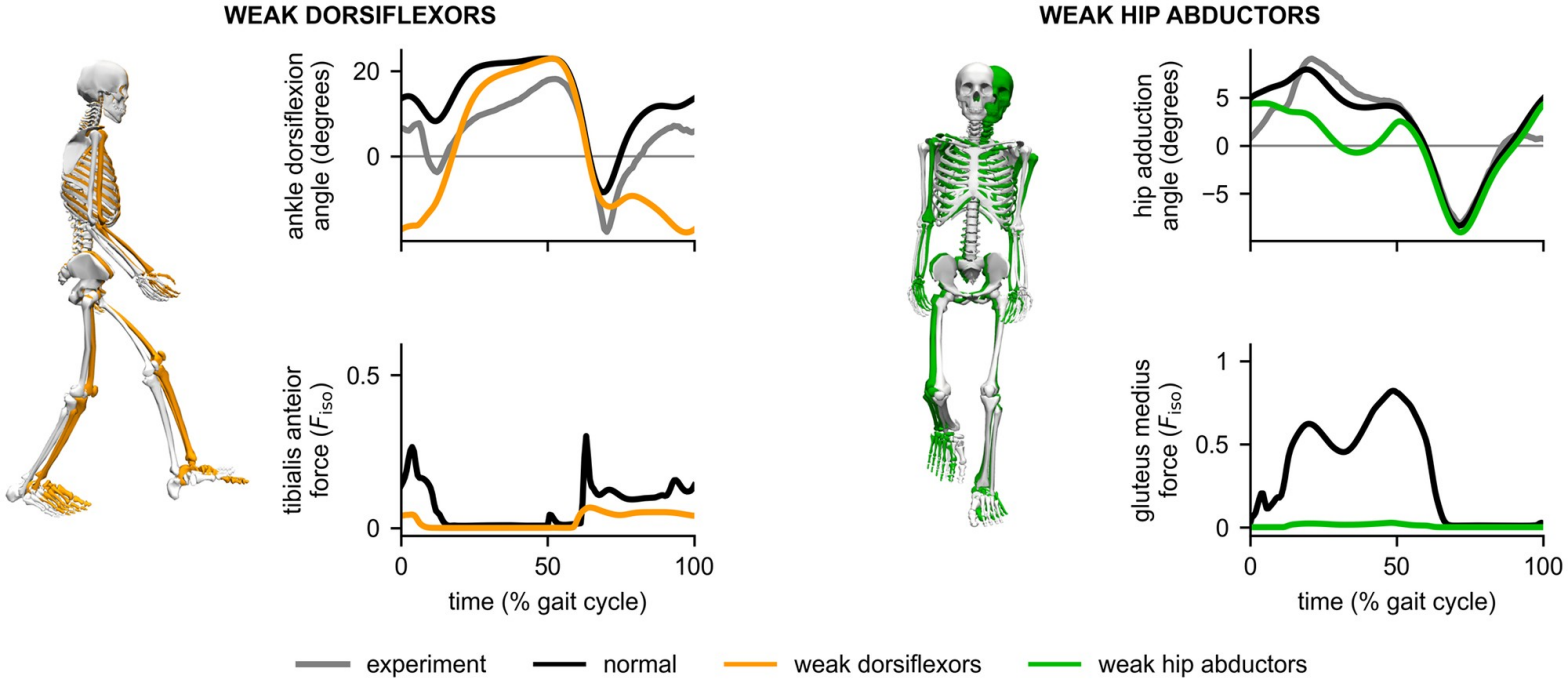
Effect of weakened dorsiflexors and hip abductors on motion tracking. Left: Weakening the ankle dorsiflexors caused ankle plantarflexion to increase during early stance and swing (orange) compared to the experimental ankle angle (gray) and the normal tracking solution (black curves, white skeleton). The tibialis anterior force is normalized by the normal max isometric force of the muscle, *F*_iso_. Right: Weakening the hip abductors resulted in a reduced hip adduction angle (green) during stance compared to the experimental hip adduction angle (gray) and the normal tracking solution (black). As shown in the skeleton graphic, increased trunk sway was observed (green) compared to the normal tracking solution (white). The force in the gluteus medius, a primary hip abductor, was nearly reduced to zero across the gait cycle.

We next weakened the ankle dorsiflexor muscles (tibialis anterior, extensor digitorum longus, and extensor hallucis longus) by reducing max isometric forces by 95% and solved the tracking problem again (using the “normal” solution for the initial guess). *MocoTrack* produced a “drop foot” [68] walking solution, where the model was unable to dorsiflex the ankle in early stance and in swing (Fig 9, left). We solved the problem again after restoring the dorsiflexor muscle strengths and weakening the hip abductor muscles (gluteus medius, gluteus minimus, and tensor fascia lata) by 90% (using the “normal” solution for the initial guess). For both weakened conditions, we used the original cost terms that penalized squared controls and tracking error. While such tracking problems are rarely used to predict gait adaptations, they provide a compromise between accuracy and the careful research required to “design” cost terms that reproduce walking without tracking data. Since major hip abductor forces were nearly reduced to zero (e.g., gluteus medius), the model was unable to produce the hip adduction angle observed in the experimental data and normal tracking solution. The adapted gait included a large increase in truck sway (Fig 9, right), which is characteristic of Trendelenburg gait [43, 68]. *MocoTrack* solved the weakened dorsiflexor and hip abductor problems in 101 and 148 minutes, respectively.

### Predicting and assisting a squat-to-stand motion

**Fig 10:**
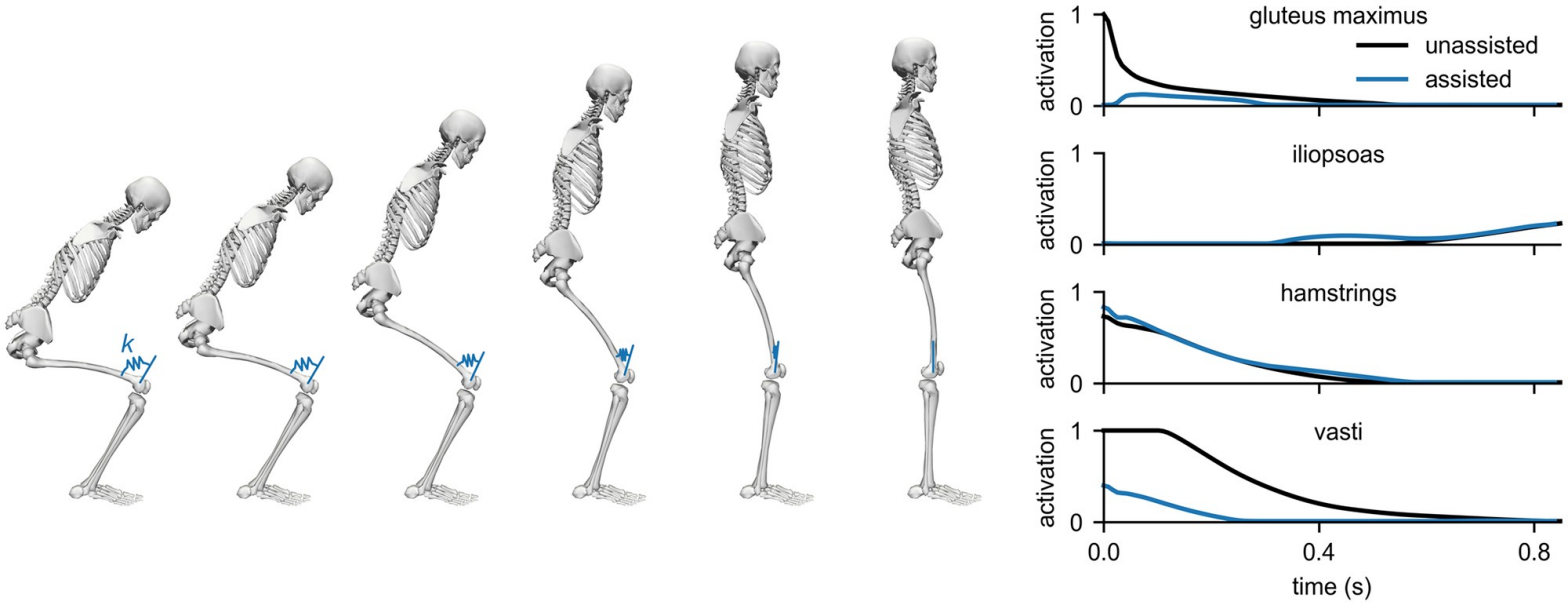
Predicting and assisting a squat-to-stand motion. Left: We predicted a squat-to-stand motion with prescribed initial and final poses that minimized the sum of squared muscle excitations and final time, then added a torsional spring to the knee and solved for the optimal spring stiffness *k*. Right: The activations of key muscles throughout the motion without (black) and with (blue) the assistive spring show that the spring allowed gluteus maximus and vasti activity to decrease substantially.

To show that Moco can predict motions and optimize parameters of a model, we predicted a squat-to-stand motion that minimized a combination of muscle effort, expressed as the sum of squared excitations (weight: 1.0), and the duration of the motion (weight: 1.0). The initial pose was prescribed to be squatting [74], and the final pose was prescribed to be upright standing. No motion was tracked. The model contained a torso and a single leg actuated by 9 muscles with activation dynamics; 6 of these muscles had compliant tendon dynamics (hamstrings, rectus femoris, vasti, gastrocnemius, soleus, and tibialis anterior). Muscle strengths were taken from a previous study [12] and doubled to model both legs as one. We modeled foot–ground contact by fixing the foot of the model to the ground with a weld joint. The model contained the same kinematic constraints dictating patellar motion as described in the previous result. The initial guess for the kinematic variables was the midpoint of the bounds on the variables; the initial guess for muscle excitations and activations was 0.05. The predicted motion and muscle activations are shown in Fig 10 (black lines). As expected [75], extensor muscles such as the gluteus maximus, hamstrings, and vasti exhibited substantial activity.

Next, we added a torsional spring to the knee and solved for the optimal motion, muscle activations, and spring stiffness. The spring was in equilibrium when the knee was extended, as illustrated in Fig 10. We used the same cost as in the unassisted case; no motion was tracked. With the assistive device, the motion was achieved with lower muscle activity. The optimal spring stiffness was 90 N-m/rad.

Both predictions converged in 3 minutes. The ability to rapidly predict motions and optimize device parameters makes Moco a valuable tool for designing assistive devices.

The duration of the various optimizations presented in this section are summarized in Table 1.

**Table 1:** Durations and settings for optimization problems.

problem	duration (minutes)	# mesh intervals	# DOFs	# muscles	convergence tolerance	constraint tolerance
MocoInverse, gait, normal (Fig 7, black)	3.5	25	29	80	10^−2^	10^−3^
MocoInverse, gait, minimizing knee load (Fig 7, blue)	15	25	29	80	10^−2^	10^−3^
MocoTrack, gait, normal (Fig 8)	130	40	29	80	10^−3^	10^−3^
MocoTrack, gait, weak dorsiflexors (Fig 9, orange)	101	40	29	80	10^−3^	10^−3^
MocoTrack, gait, weak hip abductors (Fig 9, green)	148	40	29	80	10^−3^	10^−3^
Predictive MocoStudy, squat-to-stand, unassisted (Fig 10, black)	3	50	4	9	10^−3^	10^−3^
Predictive MocoStudy, squat-to-stand, assisted (Fig 10, blue)	3	50	4	9	10^−3^	10^−3^

Durations were obtained with a 3.6 GHz Intel Core i7 processor with 8 parallel threads. All problems were solved with the Hermite–Simpson transcription scheme; each mesh interval in this scheme contains two points on the interval boundary and a collocation point at the interval midpoint (i.e., a problem with *N* mesh intervals has 2*N* + 1 time points). DOFs: degrees of freedom; convergence tolerance: the tolerance on the KKT conditions [20]; constraint tolerance: the largest permissible value of any single constraint violation [63].

### Summary of verification and validation

For each of our results, we validated our simulated quantities against experimental electromyography, kinematics, and ground reaction forces as appropriate. To ensure our solutions were not sensitive to our choice of mesh, we performed a sensitivity analysis (S2 Fig). Our verification and validation shows that Moco is capable of producing meaningful results but does not guarantee that users will obtain meaningful results for their own applications. Researchers must validate the results they obtain with their own models, cost terms, constraints, and data by comparing their results to appropriate experimental data; we encourage researchers to follow previously published guidelines [66] for validation.

## Availability and future directions

OpenSim Moco is free to download for Windows and Mac on the Moco website (https://opensim.stanford.edu/moco) and SimTK (https://simtk.org/projects/opensim-moco). The source code is available on GitHub (https://github.com/opensim-org/opensim-core). The Moco source code is licensed under the permissive Apache License 2.0, though some dependencies have more restrictive licenses (e.g., CasADi [62] is available under the GNU Lesser General Public License).

The documentation for Moco contains four main sections. The User Guide explains how to use Moco and provides tips for posing a problem. The Theory Guide explains how Moco implements direct collocation. The Application Programming Interface (API) Reference describes the classes and functions in the library. The Developer Guide explains our software design. We provide a two-page “cheat sheet” that demonstrates common commands. To use Moco effectively, users must be familiar with various topics not covered in the documentation, including optimal control theory and direct collocation [20], numerical methods (e.g., finite differences) and optimization and associated pitfalls (e.g., local minima), multibody dynamics, Hill-type muscle models [26], and musculoskeletal modeling in OpenSim [54].

Moco contains examples in MATLAB, Python, and C++ that range from predicting the optimal trajectory for a double pendulum to predicting 2-D muscle-driven walking (which solves in 30 minutes). The code that generated the results for this paper is available at https://github.com/stanfordnmbl/mocopaper.

Moco and the direct collocation method have a number of limitations that should be considered when planning a simulation study. Moco currently lacks the ability to handle certain optimal control problems, such as those with multiple phases [45] or unilateral kinematic constraints [76]. Bringing custom model components (e.g., a fatigable muscle) into Moco requires knowledge of C++ and the OpenSim software architecture; future versions of the software should allow users to implement custom model components in MATLAB or Python. The performance and ease of use of the direct collocation solvers in Moco could be improved. Computing the nonlinear program derivatives with algorithmic differentiation instead of finite differences would vastly improve the speed of Moco [50]. Supporting mesh refinement would allow the solver to increase the number of mesh intervals in time ranges with fast dynamics, thereby improving accuracy ([20], section 4.7). Moco attempts to produce well-scaled optimization problems to facilitate rapid convergence (e.g., using normalized tendon force as a state variable), but providing automated problem scaling ([20], section 4.8) could further improve convergence. Lastly, the direct collocation method itself is not well-suited to simulations that are interactive (e.g., users perturbing the model) or include randomness (e.g., uneven terrain); such problems are better suited to single shooting [12] or reinforcement learning approaches [77].

Going forward, we will focus on applying Moco to high-impact topics in biomechanics, including orthopedic surgeries and rehabilitation strategies such as muscle strength training. Predictions of movement typically employ generic models because large-scale subject-specific simulations are difficult to build. Moco permits researchers to easily build and share subject-specific studies that could discern which individuals may benefit from an intervention. Using Moco with data from inertial measurement units [78] or videos processed with computer vision [79] will allow researchers to study muscle behavior for movements based on observations outside of the laboratory.

The Moco project benefits from an active community of researchers employing musculoskeletal simulation. We have engaged this community by hosting workshops on Moco at international conferences, at Stanford University, and online. We will continue to engage this community through additional workshops and by helping others to contribute documentation, examples, teaching materials, and code.

We designed Moco to be easy to use, customizable, and extensible. We verified the software and applied it to multiple musculoskeletal problems. Moco handles models with kinematic constraints, muscle activation dynamics, compliant tendons, and compliant contact, and can minimize a combination of complex costs such as marker tracking and joint reaction loads. Given this foundation, we expect Moco to accelerate research by reducing the time spent wrestling with simulation tools and enabling our field to tackle more ambitious problems.

## Supporting information

S1 Appendix Mathematical details. This appendix describes in detail how Moco solves optimal control problems with direct collocation.
(PDF)

S1 Fig Comparing MocoInverse to static optimization and computed muscle control. The OpenSim Static Optimization and Computed Muscle Control tools produced muscle activations with similar timing as those produced by *MocoInverse* for the walking motion presented in Fig 7. Across all tools, magnitudes were similar for all muscles shown except medial gastrocnemius, soleus, and tibialis anterior. Differences in magnitudes were caused by differences in the algorithms, such as how tendon compliance was handled.
(TIF)

S2 Fig Convergence analysis. To ensure our results were not sensitive to the chosen number of mesh intervals (which is related to mesh density), we performed a convergence analysis: we ensured that the objective function value converged on a single value as the number of mesh intervals increased. We performed the convergence analysis for three problems from the results: “MocoInverse, gait, normal” (Fig 7, black), “MocoTrack, gait, normal” (Fig 8), and “Predictive MocoStudy, squat-to-stand, unassisted” (Fig 10, black). For each graph, the objective is normalized by the value of the objective from using the greatest number of mesh intervals.
(TIF)
