# Supplementary material for: OpenSim Moco: Musculoskeletal optimal control
Source: PLoS Comput Biol. 2020 Dec 28;16(12):e1008493. doi: 10.1371/journal.pcbi.1008493 (PMC7793308; doi:10.1371/journal.pcbi.1008493)
Supplement: S1 Appendix — This appendix describes in detail how Moco solves optimal control problems with direct collocation. (PDF) [file pcbi.1008493.s001.pdf]

OpenSim Moco: Musculoskeletal optimal control

S1 Appendix

Christopher L. Dembia[1☯], Nicholas A. Bianco[1☯], Antoine Falisse[2], Jennifer L. Hicks[3], Scott L. Delp[1,3,4]

1 Department of Mechanical Engineering, Stanford University, Stanford, California, United States of America
2 Department of Movement Sciences, KU Leuven, Leuven, Belgium
3 Department of Bioengineering, Stanford University, Stanford, California, United States of America
4 Department of Orthopaedic Surgery, Stanford University, Stanford, California, United States of America

☯These authors contributed equally to this work.

Moco's optimal control problem

Moco poses the optimal control problem shown in Eq 1. We seek the time-dependent states $y(t)$ and controls $x(t)$ that minimize a sum of costs J_j with weights w_j. The states include generalized coordinates $q(t)$, generalized speeds $u(t)$, and auxiliary states $z(t)$, such as muscle activations. We may also seek time-invariant parameters p, the initial time of the motion t_0, or the final time of the motion t_f. We place lower (L) and upper (U) bounds on t_0, t_f, $y(t)$, $x(t)$, and p. Additionally, we place lower and upper bounds on the initial and final values of the states and controls; this permits solving problems with prescribed initial and final states such as standing from a squat.

$$
\begin{aligned}
\text{minimize} \quad & \sum_j w_j J_j(t_0, t_f, y_0, y_f, x_0, x_f, \lambda_0, \lambda_f, p, S_{c,j}) && \text{costs} \\
& \quad S_{c,j} = \int_{t_0}^{t_f} s_{c,j}(t, y, x, \lambda, p)\, dt && \\
\text{subject to} \quad & \dot{q} = u && \\
& M(q,p)\dot{u} + G(q,p)^T \lambda = f_{\text{app}}(t, y, x, p) - f_{\text{inertial}}(q, u, p) && \text{multibody dynamics} \\
& \dot{z}_{\text{ex}}(t) = f_{\dot{z},\text{ex}}(t, y, x, \lambda, p) && \text{auxiliary dynamics, explicit} \\
& 0 = f_{\dot{z},\text{im}}(t, y, \dot{z}_{\text{im}}, x, \lambda, p) && \text{auxiliary dynamics, implicit} \\
& 0 = \phi(q,p) && \text{kinematic constraints} \\
& V_{L,k} \leq V_k(t_0, t_f, y_0, y_f, x_0, x_f, \lambda_0, \lambda_f, p, S_{b,k}) \leq V_{U,k} && \text{boundary constraints} \\
& \quad S_{b,k} = \int_{t_0}^{t_f} s_{b,k}(t, y, x, \lambda, p)\, dt \quad k = 1, \ldots, K && \\
& g_L \leq g(t, y, x, \lambda, p) \leq g_U && \text{path constraints} \\
& y_{0,L} \leq y_0 \leq y_{0,U} \qquad y_{f,L} \leq y_f \leq y_{f,U} && \text{initial and final states} \\
& x_{0,L} \leq x_0 \leq x_{0,U} \qquad x_{f,L} \leq x_f \leq x_{f,U} && \text{initial and final controls} \\
\text{with respect to} \quad & t_0 \in [t_{0,L}, t_{0,U}] && \text{initial time} \\
& t_f \in [t_{f,L}, t_{f,U}] && \text{final time} \\
& y(t) = (q(t), u(t), z(t)) \in [y_L, y_U] && \text{states} \\
& x(t) \in [x_L, x_U] && \text{controls} \\
& \lambda(t) && \text{Lagrange multipliers} \\
& p \in [p_L, p_U] && \text{time-invariant parameters}
\end{aligned}
\tag{1}
$$

The problem contains constraints for the system's multibody dynamics (involving the mass matrix M; applied forces f_{app} from gravity, muscles, etc.; and centripetal and Coriolis terms f_{inertial}) and any auxiliary dynamics. The auxiliary dynamics may be expressed as explicit ($f_{\dot{z},\text{ex}}$) or implicit ($f_{\dot{z},\text{im}}$) differential equations; the auxiliary state variables z are partitioned according to whether they are integrated using explicit differential equations (z_{ex}) or implicit differential equations (z_{im}). The system may contain position-level (holonomic) kinematic constraints to, for example, weld a foot to a bicycle pedal. Each constraint is enforced by forces exerted by tissue, bones, bodies, or other parts of the modeled system. These generalized constraint forces are applied in the constrained directions (e.g., the six degrees of freedom between the foot and pedal), and we introduce time-varying Lagrange multiplier variables λ to solve for these forces. The derivatives of the kinematic constraints ϕ with respect to the generalized coordinates yields the kinematic constraint Jacobian G; the transpose of this matrix converts the Lagrange multipliers into generalized forces along the system's degrees of freedom. See below for details on how Moco handles kinematic constraints.

Additionally, the problem contains K boundary constraints V_k (with bounds $V_{L,k}$ and $V_{U,k}$) and algebraic path constraints g over the motion (with time-invariant bounds g_L and g_U). The cost terms and boundary constraints may depend on initial and final time; states; controls; kinematic constraint multipliers (required for joint reactions); time-invariant parameters; and an integral, $S_{c,j}$ or $S_{b,k}$, over the motion.

Kinematic constraints

Support for problems incorporating kinematic constraints is a key feature of Moco. Understanding how kinematic constraints are handled in multibody dynamics is valuable for understanding how Moco handles such problems. Consider a two-dimensional point mass system with coordinates q_x and q_y constrained to a parabola $0 = q_y - q_x^2$. To prevent the point mass from violating the constraint, we must apply a force perpendicular to the parabola. Each constraint has a corresponding scalar force variable, called a Lagrange multiplier λ. We must solve for the required magnitudes of these constraint forces, but the direction in which we apply each of these forces is determined by the derivative of the constraint equations. We gather the derivatives of the constraint equations in the kinematic constraint Jacobian matrix G. Each row in this matrix contains the derivative of a single constraint equation with respect to each degree of freedom, and the matrix has a column for each degree of freedom. For the parabola example, the Jacobian is $(-2q_x, 1)$. The transpose of this matrix, G^T, contains columns that are vectors in the state space which are perpendicular to each constraint. For our single constraint, the vector $(-2q_x, 1)^T$ is perpendicular to the parabola. The matrix-vector product between the Jacobian transpose and the Lagrange multipliers, $G^T\lambda$, yields the vector of generalized forces (whose length is the number of degrees of freedom) necessary for enforcing the kinematic constraints. For our point mass example, the generalized forces yielded by the vector-scalar product $(-2q_x, 1)^T\lambda$ keep the point mass on the parabola. To apply these forces to the multibody system, we include the $G^T\lambda$ term in the multibody dynamics equations of motion.

When simulating a multibody system with time-stepping forward integration, we first ensure the initial generalized coordinates and speeds satisfy the kinematic constraints $\phi(q) = 0$ and their first derivative $\dot{\phi}(q,u) = 0$ via a root-solve. During the integration, we solve for generalized accelerations and Lagrange multipliers that obey the multibody dynamics equations of motion and the second derivative of the kinematic constraints, $\ddot{\phi}(q,u,\dot{u}) = 0$. Numerically integrating the resulting generalized accelerations yields generalized coordinates and speeds that approximately lie on the constraint manifold defined by $\phi(q) = 0$; to fix any errors in the constraints caused by numerical integration error, we project the generalized coordinates and speeds back onto the constraint manifold [1].

In direct collocation, we solve for the entire trajectory of the system—including the generalized coordinates, generalized speeds, and Lagrange multipliers—all at once. When expressing multibody dynamics as implicit differential equations, the generalized accelerations are also unknowns. How we solve for the trajectory of the system in the presence of kinematic constraints depends on the transcription scheme; see the remainder of this Appendix for details.

Moco's optimal control problem with prescribed kinematics

A common task in musculoskeletal biomechanics is to estimate the muscle and actuator behavior that drove an observed motion. We can solve this problem by minimizing the error between the observed motion and the simulated motion, as with Computed Muscle Control (using the "slow target") [2] or *MocoTrack*. Alternatively, we can prescribe the motion exactly, as with Static Optimization [3], electromyography-driven simulation [4], and the Muscle Redundancy Solver [5]. Consider a two-dimensional point mass with coordinates q_x and q_y for which we prescribe a circular motion via the functions $\hat{q}_x(t) = \cos(t)$ and $\hat{q}_y(t) = \sin(t)$. We can either add these functions to the kinematic constraints $\phi(q)$, or we can substitute these functions into the equations of motion, thereby eliminating the variables q_x and q_y. With Moco, users can choose either the former approach through OpenSim's *Coordinate*, or the latter (and usually preferable) approach using *PositionMotion*, a new component that employs Simbody's *Motion* class. Prescribing kinematics by eliminating variables leads to a problem that is robust and fast—the nonlinear multibody dynamics are removed from

the optimization problem—but prevents predicting kinematic deviations from the observed motion.

When we prescribe kinematics in Moco by eliminating variables, we replace the problem in Eq 1 with the following:

$$
\begin{array}{llll}
\text{minimize} & \displaystyle\sum_j w_j J_j(t_0, t_f, \hat{q}_0, \hat{q}_f, \hat{u}_0, \hat{u}_f, z_0, z_f, x_0, x_f, \lambda_0, \lambda_f, p, S_{c,j}) & & \text{costs} \\
& \qquad S_{c,j} = \displaystyle\int_{t_0}^{t_f} s_{c,j}(t, \hat{q}, \hat{u}, z, x, \lambda, p)\, dt & & \\
\text{subject to} & M(\hat{q}, p)\dot{\hat{u}} + G(\hat{q}, p)^T \lambda = f_{\text{app}}(t, \hat{q}, \hat{u}, z, x, p) - f_{\text{inertial}}(\hat{q}, \hat{u}, p) & & \text{multibody dynamics} \\
& \dot{z}_{\text{ex}}(t) = f_{\dot{z},\text{ex}}(t, \hat{q}, \hat{u}, z, x, \lambda, p) & & \text{auxiliary dynamics, explicit} \\
& 0 = f_{\dot{z},\text{im}}(t, \hat{q}, \hat{u}, z, \dot{z}_{\text{im}}, x, \lambda, p) & & \text{auxiliary dynamics, implicit} \\
& V_{L,k} \le V_k(t_0, t_f, \hat{q}_0, \hat{q}_f, \hat{u}_0, \hat{u}_f, z_0, z_f, x_0, x_f, \lambda_0, \lambda_f, p, S_{b,k}) \le V_{U,k} & & \text{boundary constraints} \\
& \qquad S_{b,k} = \displaystyle\int_{t_0}^{t_f} s_{b,k}(t, \hat{q}, \hat{u}, z, x, \lambda, p)\, dt \quad k = 1, \ldots, K & & \\
& g_L \le g(t, \hat{q}, \hat{u}, z, x, \lambda, p) \le g_U & & \text{path constraints} \\
& z_{0,L} \le z_0 \le z_{0,U} \qquad z_{f,L} \le z_f \le z_{f,U} & & \text{initial and final states} \\
& x_{0,L} \le x_0 \le x_{0,U} \qquad x_{f,L} \le x_f \le x_{f,U} & & \text{initial and final controls} \\
\text{with respect to} & t_0 \in [t_{0,L}, t_{0,U}] \qquad \text{initial time} & & \\
& t_f \in [t_{f,L}, t_{f,U}] \qquad \text{final time} & & \\
& z(t) \in [z_L, z_U] \qquad \text{auxiliary states} & & \\
& x(t) \in [x_L, x_U] \qquad \text{controls} & & \\
& \lambda(t) \qquad \text{Lagrange multipliers} & & \\
& p \in [p_L, p_U]. \qquad \text{time-invariant parameters} & &
\end{array}
\tag{2}
$$

We replace the kinematic variables q and u with known quantities $\hat{q}$ and $\hat{u}$. The system still depends on auxiliary state variables z and control variables x, and includes auxiliary dynamics. If none of the parameter variables affect the multibody system, then the multibody dynamics are reduced to a force balance: muscles and other force elements must generate the net generalized forces determined by the kinematics and external loads data.

Whether the motion is prescribed by adding constraints or eliminating variables, OpenSim supplements the modeled force elements with Lagrange multipliers to ensure the prescribed motion is achieved. When using *PositionMotion* with Moco, we require that the prescribed motion's Lagrange multipliers are zero, thereby ensuring the motion is fully generated by the modeled force elements. The easiest way to prescribe kinematics in Moco is to use the *MocoInverse* tool, which uses *PositionMotion* internally.

Transcription schemes

Trapezoidal transcription

The trapezoidal scheme transcribes the optimal control problem into a nonlinear program by approximating integrals using the trapezoidal rule. As a second-order scheme, trapezoidal transcription exhibits accuracy that improves four-fold when halving the mesh interval (i.e., time step).

We discretize the continuous variables t, y, x, and λ on a mesh of time points t_i defined

by dimensionless time τ_i, yielding n mesh intervals with durations h_i:

$$\begin{aligned} 0 = \tau_0 < \tau_1 < \tau_2 < \ldots < \tau_i < \ldots < \tau_{n-1} < \tau_n = 1, \\ t_i = (t_f - t_0)\tau_i + t_0, \\ h_i = (t_f - t_0)(\tau_i - \tau_{i-1}). \end{aligned} \tag{3}$$

For conciseness, we define the following function:

$$\text{trap}_i(F(\eta, p)) = \frac{1}{2} h_i (F(\eta_{i-1}, p) + F(\eta_i, p)), \tag{4}$$

where $\text{trap}_i(F(\eta, p)))$ is a trapezoidal rule approximation of the area under the function F for mesh interval i, and η represents any subset of continuous variables.

We define the kinematic and dynamic explicit differential equations as:

$$f_{\dot{q}}(u) = u \tag{5}$$

$$f_{\dot{u}}(t, y, x, \lambda, p) = M(q, p)^{-1} \big[(f_{\text{app}}(t, y, x, p) - f_{\text{inertial}}(q, u, p) - G(q, p)^T \lambda \big]. \tag{6}$$

The mass matrix M, the centripetal and Coriolis forces f_{inertial}, and kinematic constraint Jacobian G are computed by Simbody (OpenSim's multibody dynamics engine) using order-N recursive algorithms[1] [1]. The applied forces f_{app} can include any force elements supported by Simbody, and are often defined by OpenSim components that provide access to existing Simbody force elements (e.g., *SmoothSphereHalfSpaceForce*) or that define custom Simbody force elements (e.g., *DeGrooteFregly2016Muscle*). Simbody computes the constraint Jacobian G based on any Simbody kinematic constraints that the OpenSim model adds to the system. In Moco, the Lagrange multipliers λ are explicit optimization variables; this approach differs from that in time-stepping forward integrations, in which Simbody solves for generalized accelerations and Lagrange multipliers simultaneously.

The result of the trapezoidal transcription, with multibody dynamics expressed as explicit

[1] In contrast to software based on hand-written or symbolically-derived equations of motion, Simbody does not compute the complete mass matrix explicitly.

differential equations, is the following nonlinear program:

$$
\begin{aligned}
\text{minimize} \quad & \sum_j w_j J_j(t_0, t_f, y_0, y_n, x_0, x_n, \lambda_0, \lambda_n, p, S_{c,j}) + w_\lambda \sum_{i=1}^{n} \text{trap}_i(\|\lambda\|_2^2) \\
& \quad S_{c,j} = \sum_{i=1}^{n} \text{trap}_i(s_{c,j}(t, y, x, \lambda, p)) \\
\text{subject to} \quad & q_i = q_{i-1} + \text{trap}_i(f_{\dot{q}}(u)) && i = 1, \ldots, n \\
& u_i = u_{i-1} + \text{trap}_i(f_{\dot{u}}(t, y, x, \lambda, p)) && i = 1, \ldots, n \\
& z_{\text{ex},i} = z_{\text{ex},i-1} + \text{trap}_i(f_{\dot{z},\text{ex}}(t, y, x, \lambda, p)) && i = 1, \ldots, n \\
& z_{\text{im},i} = z_{\text{im},i-1} + \text{trap}_i(\zeta) && i = 1, \ldots, n \\
& 0 = f_{\dot{z},\text{im}}(t_i, y_i, \zeta_i, x_i, \lambda_i, p) && i = 0, \ldots, n \\
& 0 = \phi(q_i, p) && i = 0, \ldots, n \\
& V_{L,k} \le V_k(t_0, t_f, y_0, y_f, x_0, x_f, \lambda_0, \lambda_f, p, S_{b,k}) \le V_{U,k} \\
& \quad S_{b,k} = \sum_{i=1}^{n} \text{trap}_i(s_{b,k}(t, y, x, \lambda, p)) && k = 1, \ldots, K \\
& g_L \le g(t_i, y_i, x_i, \lambda_i, p) \le g_U && i = 0, \ldots, n \\
\text{with respect to} \quad & t_0 \in [t_{0,L}, t_{0,U}] \qquad t_n \in [t_{f,L}, t_{f,U}] \\
& y_0 \in [y_{0,L}, y_{0,U}] \qquad y_n \in [y_{f,L}, y_{f,U}] \\
& x_0 \in [x_{0,L}, x_{0,U}] \qquad x_n \in [x_{f,L}, x_{f,U}] \\
& y_i \in [y_L, y_U] && i = 1, \ldots, n-1 \\
& x_i \in [x_L, x_U] && i = 1, \ldots, n-1 \\
& \zeta_i \in [\zeta_L, \zeta_U] && i = 0, \ldots, n \\
& \lambda_i \in [\lambda_L, \lambda_U] && i = 0, \ldots, n \\
& p \in [p_L, p_U].
\end{aligned}
\tag{7}
$$

In this form, the problem can be solved directly by a nonlinear program solver. We introduce the algebraic (control) variable ζ as the derivative of auxiliary state variables whose dynamics are expressed with an implicit differential equation.

When expressing the multibody dynamics implicitly, we remove the constraint involving $f_{\dot{u}}$, introduce generalized accelerations as an algebraic variable υ and enforce multibody dynamics in "inverse dynamics" form:

$$
\begin{aligned}
\text{subject to} \quad & u_i = u_{i-1} + \text{trap}_i(\upsilon) && i = 1, \ldots, n \\
& M(q_i, p)\upsilon_i + G(q_i, p)^T \lambda_i = f_{\text{app},i} - f_{\text{inertial},i} && i = 0, \ldots, n \\
\text{with respect to} \quad & \upsilon_i \in [-\upsilon_B, \upsilon_B] && i = 0, \ldots, n.
\end{aligned}
\tag{8}
$$

The constant υ_B is a large positive number (1000 by default).

The dynamic, kinematic, and path constraints are enforced at a set of discrete time points, so the quadratic spline approximation to the continuous variables may violate the original continuous-time constraints between the discrete time points. For this reason, a mesh with more points leads to a more accurate solution.

Our implementation of trapezoidal transcription handles kinematic constraints, but not in the most robust fashion. We enforce ϕ but not its time derivatives; enforcing the constraints at only the position level yields an index-3 system of differential-algebraic equations, which are challenging to solve [6–8] (the differential-algebraic equations are "index-3" because the algebraic constraints ϕ must be differentiated three times to convert the system of differential-algebraic equations into ordinary differential equations). To improve numerical

conditioning, we minimize the Lagrange multipliers associated with the constraints (with weight w_λ; see Eq 7).

When kinematics are prescribed (see "Moco's optimal control problem with prescribed kinematics"), multibody dynamics must be expressed implicitly and kinematic constraints are not enforced; we expect the prescribed kinematics to already obey the constraints.

Hermite-Simpson transcription

The Hermite-Simpson scheme transcribes the optimal control problem into a nonlinear program by approximating integrals using a Hermite interpolant and Simpson integration rule. As a third-order scheme, Hermite-Simpson transcription exhibits accuracy that improves eight-fold when halving the mesh interval.

We use a similar dimensionless time mesh as for the trapezoidal scheme, with n mesh intervals with durations h_i. We also introduce collocation points at the midpoints of the mesh intervals, leading to a total of $2n+1$ time points at which we discretize the continuous variables:

$$\begin{aligned} 0 = \tau_0 < \tau_1 < \tau_2 < \ldots < \tau_i < \ldots < \tau_{n-1} < \tau_n = 1, \\ \bar{\tau}_i = 0.5(\tau_{i-1} + \tau_i), \\ t_i = (t_f - t_0)\tau_i + t_0, \\ \bar{t}_i = (t_f - t_0)\bar{\tau}_i + t_0, \\ h_i = (t_f - t_0)(\tau_i - \tau_{i-1}), \end{aligned} \tag{9}$$

where $\bar{\tau}_i$ denote mesh interval midpoints.

For conciseness, we define the following functions:

$$\text{hermite}_i(\eta, F(\eta, p)) = \frac{1}{2}(\eta_{i-1} + \eta_i) + \frac{h_i}{8}\big(F(\eta_{i-1}, p) - F(\eta_i, p)\big), \tag{10}$$

$$\text{simpson}_i(F(\eta, p)) = \frac{h_i}{6}\big(F(\eta_{i-1}, p) + 4F(\bar{\eta}_i, p) + F(\eta_i, p)\big), \tag{11}$$

where $\text{hermite}_i()$ represents the Hermite interpolant, which yields the value of the midpoint of a segment in a Hermite spline, and $\text{simpson}_i()$ represents the Simpson integration rule. Again, F is a function for mesh interval i, and η represents any subset of continuous variables. The symbols $\bar{\eta}_i$ represent continuous variables evaluated at the mesh interval midpoints.

Using the explicit multibody dynamics function $f_{\dot{u}}$ defined previously, Hermite-Simpson

transcription results in the following nonlinear program:

$$
\begin{aligned}
\text{minimize} \quad & \sum_j w_j J_j(t_0, t_f, y_0, y_n, x_0, x_n, \lambda_0, \lambda_n, p, S_{c,j}) + w_\lambda \sum_{i=1}^{n} \text{simpson}_i(\|\lambda\|_2^2) \\
& \qquad S_{c,j} = \sum_{i=1}^{n} \text{simpson}_i(s_{c,j}(t, y, x, \lambda, p)) \\
\text{subject to} \quad & \bar{q}_i = \text{hermite}_i(q, f_{\dot{q}}(q, u, \gamma, p)) && i = 1, \ldots, n \\
& q_i = q_{i-1} + \text{simpson}_i(f_{\dot{q}}(q, u, \gamma, p)) && i = 1, \ldots, n \\
& \bar{u}_i = \text{hermite}_i(u, f_{\dot{u}}(t, y, x, \lambda, p)) && i = 1, \ldots, n \\
& u_i = u_{i-1} + \text{simpson}_i(f_{\dot{u}}(t, y, x, \lambda, p)) && i = 1, \ldots, n \\
& \bar{z}_{\text{ex},i} = \text{hermite}_i(z_{\text{ex}}, f_{\dot{z},\text{ex}}(t, y, x, \lambda, p)) && i = 1, \ldots, n \\
& z_{\text{ex},i} = z_{\text{ex},i-1} + \text{simpson}_i(f_{\dot{z},\text{ex}}(t, y, x, \lambda, p)) && i = 1, \ldots, n \\
& \bar{z}_{\text{im},i} = \text{hermite}_i(z_{\text{im}}, \zeta) && i = 1, \ldots, n \\
& z_{\text{im},i} = z_{\text{im},i-1} + \text{simpson}_i(\zeta) && i = 1, \ldots, n \\
& 0 = f_{\dot{z},\text{im}}(t_i, y_i, \zeta_i, x_i, \lambda_i, p) && i = 0, \ldots, n \\
& \bar{x}_i = (x_{i-1} + x_i)/2 && i = 1, \ldots, n \\
& 0 = \phi(q_i, p) = \dot{\phi}(q_i, u_i, p) = \ddot{\phi}(t_i, y_i, x_i, \lambda_i, p) && i = 0, \ldots, n \\
& V_{L,k} \le V_k(t_0, t_f, y_0, y_f, x_0, x_f, \lambda_0, \lambda_f, p, S_{b,k}) \le V_{U,k} \\
& \qquad S_{b,k} = \sum_{i=1}^{n} \text{simpson}_i(s_{b,k}(t, y, x, \lambda, p)) && k = 1, \ldots, K \\
& g_L \le g(t_i, y_i, x_i, \lambda_i, p) \le g_U && i = 0, \ldots, n \\
\text{with respect to} \quad & t_0 \in [t_{0,L}, t_{0,U}] \qquad t_n \in [t_{f,L}, t_{f,U}] \\
& y_0 \in [y_{0,L}, y_{0,U}] \qquad y_n \in [y_{f,L}, y_{f,U}] \\
& x_0 \in [x_{0,L}, x_{0,U}] \qquad x_n \in [x_{f,L}, x_{f,U}] \\
& y_i \in [y_L, y_U] && i = 1, \ldots, n-1 \\
& \bar{y}_i \in [y_L, y_U] && i = 1, \ldots, n \\
& x_i \in [x_L, x_U] && i = 1, \ldots, n-1 \\
& \bar{x}_i \in [x_L, x_U] && i = 1, \ldots, n \\
& \zeta_i \in [\zeta_L, \zeta_U] && i = 0, \ldots, n \\
& \bar{\zeta}_i \in [\zeta_L, \zeta_U] && i = 1, \ldots, n \\
& \lambda_i \in [\lambda_L, \lambda_U] && i = 0, \ldots, n \\
& \bar{\lambda}_i \in [\lambda_L, \lambda_U] && i = 1, \ldots, n \\
& \bar{\gamma}_i \in [\bar{\gamma}_L, \bar{\gamma}_U] && i = 1, \ldots, n \\
& p \in [p_L, p_U].
\end{aligned}
\tag{12}
$$

The variables $\bar{y}_i$ (including $\bar{q}_i$, $\bar{u}_i$, $\bar{z}_{\text{ex},i}$, $\bar{z}_{\text{im},i}$), $\bar{x}_i$, $\bar{\zeta}_i$, $\bar{\lambda}_i$, and $\bar{\gamma}_i$ are associated with the midpoint of mesh interval i. The midpoint states are included as explicit optimization variables $\bar{y}_i$ since we are using the separated form of Hermite-Simpson transcription [8]

Eq 12 includes the first and second time derivatives of the kinematic constraints:

$$0 = \dot{\phi}(q, u, p) = G(q, p)u, \tag{13}$$

$$0 = \ddot{\phi}(t, y, x, \lambda, p) = G(q, p)\dot{u} + \dot{G}(q, p)u = G(q, p)f_{\dot{u}}(t, y, x, \lambda, p) + \dot{G}(q, p)u. \tag{14}$$

Including these constraints reduces the index of the differential-algebraic equations from 3 to 1, which provides numerical benefits [6–8]. However, simply appending the kinematic

constraints to the unconstrained optimal control problem would overconstrain the resulting non-linear program, so we use the method for handling kinematic constraints that was introduced by Posa and colleagues [9]. In addition to the Lagrange multiplier variables we introduced for trapezoidal transcription, we introduce velocity correction variables, γ, whose multiplication with the transpose of the kinematic constraint Jacobian, $G(q,p)^T\gamma$, yields a correction to the generalized speeds that is perpendicular to the constraint manifold $\phi = 0$. We update the function $f_{\dot{q}}$ used in Eq 12 accordingly:

$$f_{\dot{q}}(q,u,\gamma,p) = u + G(q,p)^T\gamma \tag{15}$$

Without this correction, the Hermite splines cannot simultaneously satisfy both the differential equations at the mesh interval midpoints and the kinematic constraints at the mesh points. Since velocity-level kinematic constraints (Eq 13) are already enforced at the mesh points, this correction term only appears at the mesh interval midpoints (i.e., $G(\bar{q}_i,p)^T\bar{\gamma}_i$); therefore, velocity corrections at the mesh points are zero and do not appear in Eq 12. Although Simbody supports non-holonomic and acceleration-level constraints, Moco only supports holonomic (position-level) constraints.

For implicit multibody dynamics, we remove the constraints involving $f_{\dot{u}}$ and introduce generalized accelerations as algebraic variables υ to enforce multibody dynamics in "inverse dynamics" form:

$$\begin{aligned}
\text{subject to}\quad & \bar{u}_i = \text{hermite}_i(u,\upsilon) & i = 1,\ldots,n \\
& u_i = u_{i-1} + \text{simpson}_i(\upsilon) & i = 1,\ldots,n \\
& M(q_i,p)\upsilon_i + G(q_i,p)^T\lambda_i = f_{\text{app},i} - f_{\text{inertial},i} & i = 0,\ldots,n \\
& M(\bar{q}_i,p)\bar{\upsilon}_i + G(\bar{q}_i,p)^T\bar{\lambda}_i = \bar{f}_{\text{app},i} - \bar{f}_{\text{inertial},i} & i = 1,\ldots,n \\
\text{with respect to}\quad & \upsilon_i \in [-\upsilon_B, \upsilon_B] & i = 0,\ldots,n \\
& \bar{\upsilon}_i \in [-\upsilon_B, \upsilon_B] & i = 1,\ldots,n.
\end{aligned} \tag{16}$$

Automated test suite

Moco contains an automated test suite with over 80 test cases. The test suite contains four major types of tests: analytical tests, interface tests, algorithmic tests, and regression tests.

In analytical tests, we ensure Moco produces the correct solutions for problems with known analytical solutions. These tests are commonly used to verify optimal control software and for performance benchmarking [15]. We solve two such problems: the "linear tangent steering" problem described in the main article and a boundary value problem involving a set of linear first-order differential equations presented by Kirk et al. [10] (Example 5.1-1, pg. 198). In addition, we include analytical tests using the *SmoothSphereHalfSpaceForce* contact model to ensure that the smooth model produces correct forces. For example, we test that a point mass containing a contact element with dissipation comes to rest with a normal force that matches the weight of the system after being dropped from a height.

Interface tests ensure that a change made by a user though the Moco interface is reflected within the software. Any values set by the user (using a "set" method) should be returned by Moco when queried (using a "get" method). Interface tests are implemented for user-facing classes in Moco, including *MocoStudy*, *MocoProblem*, *MocoSolver*, *MocoGoal*, *MocoTrajectory*, *MocoInverse*, *MocoTrack*, and the smooth model components *DeGrooteFregly2016Muscle* and *SmoothSphereHalfSpaceForce*. For example, the *MocoGoal* class contains a setting for disabling a goal without removing it from the *MocoProblem*; we include simple tests to ensure that *MocoGoals* are indeed disabled by this setting.

Algorithmic tests check that Moco solves direct collocation problems correctly. These tests check that problems using explicit and implicit dynamics produce the same trajectory, minimizing a cost term produces the expected behavior (e.g., minimum time problems hit

the lower bound on final time), and that kinematic constraints are obeyed. For example, we check that adding a coordinate coupler constraint to a double pendulum problem produces coordinate trajectories that obey a linear relationship defined by the constraint. Algorithmic tests also encompass "self-consistency" tests. For example, using a model of a point mass hanging from a muscle, we check that tracking a reference trajectory obtained by a minimum time predictive problem produces the same controls as the original problem.

For complex problems for which there is no known exact solution, we use regression tests, which check that solutions to direct collocation problems do not regress from reference trajectories. These checks ensure that changes to the codebase do not produce any unintended effects. For example, we test that *MocoInverse* and *MocoTrack* always produce the same muscle activity given the same model and solver settings.

Moco's direct collocation solvers

Moco provides two solvers as subclasses of *MocoSolver*: *MocoCasADiSolver* uses the third-party CasADi library [11], and *MocoTropterSolver* uses a direct collocation solver we developed named Tropter. CasADi is an open-source package for algorithmic differentiation and is a bridge to nonlinear program solvers IPOPT [12], SNOPT [13], and others.

Gradient-based nonlinear program solvers require the gradient of the objective, the Jacobian of the constraints, and sometimes the Hessian of the Lagrangian [8]. To maximize computational efficiency, these derivatives are ideally computed exactly through either analytic expressions or algorithmic differentiation [11, 14]. OpenSim's main distribution does not provide exact derivatives, so we use finite differences. CasADi is an ideal library for employing direct collocation, but two limitations led us to create Tropter: CasADi did not initially support finite differences, and CasADi's open-source license is more restrictive than OpenSim's. More recent versions of CasADi support finite differences and CasADi understands the structure of the nonlinear program objective and constraint functions, allowing for potentially more efficient finite difference calculations than with Tropter, which treats the nonlinear program objective and constraints as black-box functions [15]. If OpenSim provides exact derivatives in the future, we can exploit the algorithmic differentiation modes in Tropter and CasADi [16]. Those distributing Moco as a dependency of closed-source software may prefer distributing Moco without CasADi, as CasADi's "weak copyleft" GNU Lesser General Public License 3.0 places requirements on how CasADi is redistributed.

References

1. Sherman MA, Seth A, Delp SL. Simbody: multibody dynamics for biomedical research. Procedia IUTAM. 2011;2:241–261. doi:10.1016/j.piutam.2011.04.023.
2. Thelen DG, Anderson FC, Delp SL. Generating dynamic simulations of movement using computed muscle control. Journal of Biomechanics. 2003;36:321–328. doi:10.1016/s0021-9290(02)00432-3.
3. Crowninshield RD, Brand RA. A physiologically based criterion of muscle force prediction in locomotion. Journal of Biomechanics. 1981;14(11):793 – 801. doi:https://doi.org/10.1016/0021-9290(81)90035-X.
4. Lloyd DG, Besier TF. An EMG-driven musculoskeletal model to estimate muscle forces and knee joint moments in vivo. Journal of Biomechanics. 2003;36:765–776. doi:10.1016/s0021-9290(03)00010-1.
5. De Groote F, Kinney AL, Rao AV, Fregly BJ. Evaluation of Direct Collocation Optimal Control Problem Formulations for Solving the Muscle Redundancy Problem. Annals of Biomedical Engineering. 2016;44:2922–2936. doi:10.1007/s10439-016-1591-9.

6. Hairer E, Wanner G. Solving Ordinary Differential Equations II, Stiff and Differential-Algebraic Problems. Springer; 1996.

7. Campbell S, Kunkel P. Solving higher index DAE optimal control problems. Numerical Algebra, Control and Optimization. 2016;6:447–472. doi:10.3934/naco.2016020.

8. Betts JT. Practical Methods for Optimal Control and Estimation Using Nonlinear Programming. SIAM; 2010.

9. Posa M, Tedrake R, Kuindersma S. Optimization and Stabilization of Trajectories for Constrained Dynamical Systems. 2016 IEEE International Conference on Robotics and Automation. 2016; p. 1366–1373. doi:10.1109/icra.2016.7487270.

10. Kirk DE. Optimal control theory: an introduction. Courier Corporation; 2004.

11. Andersson JAE, Gillis J, Horn G, Rawlings JB, Diehl M. CasADi: a software framework for nonlinear optimization and optimal control. Mathematical Programming Computation. 2019;11:1–36. doi:10.1007/s12532-018-0139-4.

12. Wächter A, Biegler LT. On the implementation of an interior-point filter line-search algorithm for large-scale nonlinear programming. Mathematical Programming. 2006;106:25–57. doi:10.1007/s10107-004-0559-y.

13. Gill PE, Murray W, Saunders MA. SNOPT: An SQP Algorithm for Large-Scale Constrained Optimization. SIAM Review. 2005;47:99–131. doi:10.1137/s0036144504446096.

14. Walther A, Griewank A, Vogel O. ADOL-C: Automatic Differentiation Using Operator Overloading in C++. PAMM. 2003;2:41–44. doi:10.1002/pamm.200310011.

15. Patterson MA, Rao A. Exploiting Sparsity in Direct Collocation Pseudospectral Methods for Solving Optimal Control Problems. Journal of Spacecraft and Rockets. 2013;doi:10.2514/1.a32071.

16. Falisse A, Serrancolí G, Dembia CL, Gillis J, De Groote F. Algorithmic differentiation improves the computational efficiency of OpenSim-based trajectory optimization of human movement. PLoS ONE. 2019;14:e0217730. doi:10.1371/journal.pone.0217730.
