# Supplementary figures and images for: OpenSim Moco: Musculoskeletal optimal control
Source: PLoS Comput Biol. 2020 Dec 28;16(12):e1008493. doi: 10.1371/journal.pcbi.1008493 (PMC7793308; doi:10.1371/journal.pcbi.1008493)

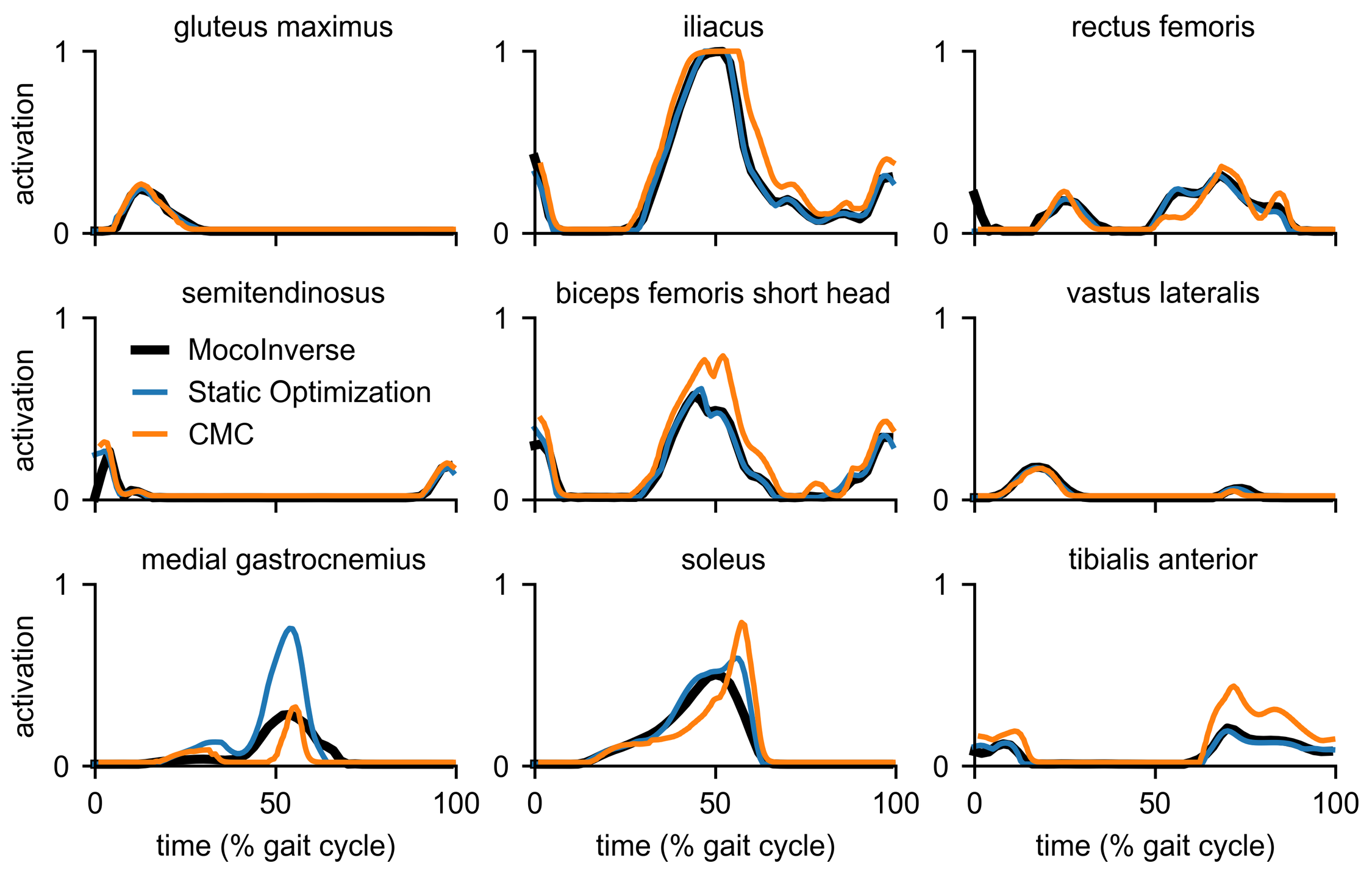

Supplement: S1 Fig — The OpenSim Static Optimization and Computed Muscle Control tools produced muscle activations with similar timing as those produced by MocoInverse for the walking motion presented in Fig 7. Across all tools, magnitudes were similar for all muscles shown except medial gastrocnemius, soleus, and tibialis anterior. Differences in magnitudes were caused by differences in the algorithms, such as how tendon compliance was handled. (TIF) [file pcbi.1008493.s002.tif]

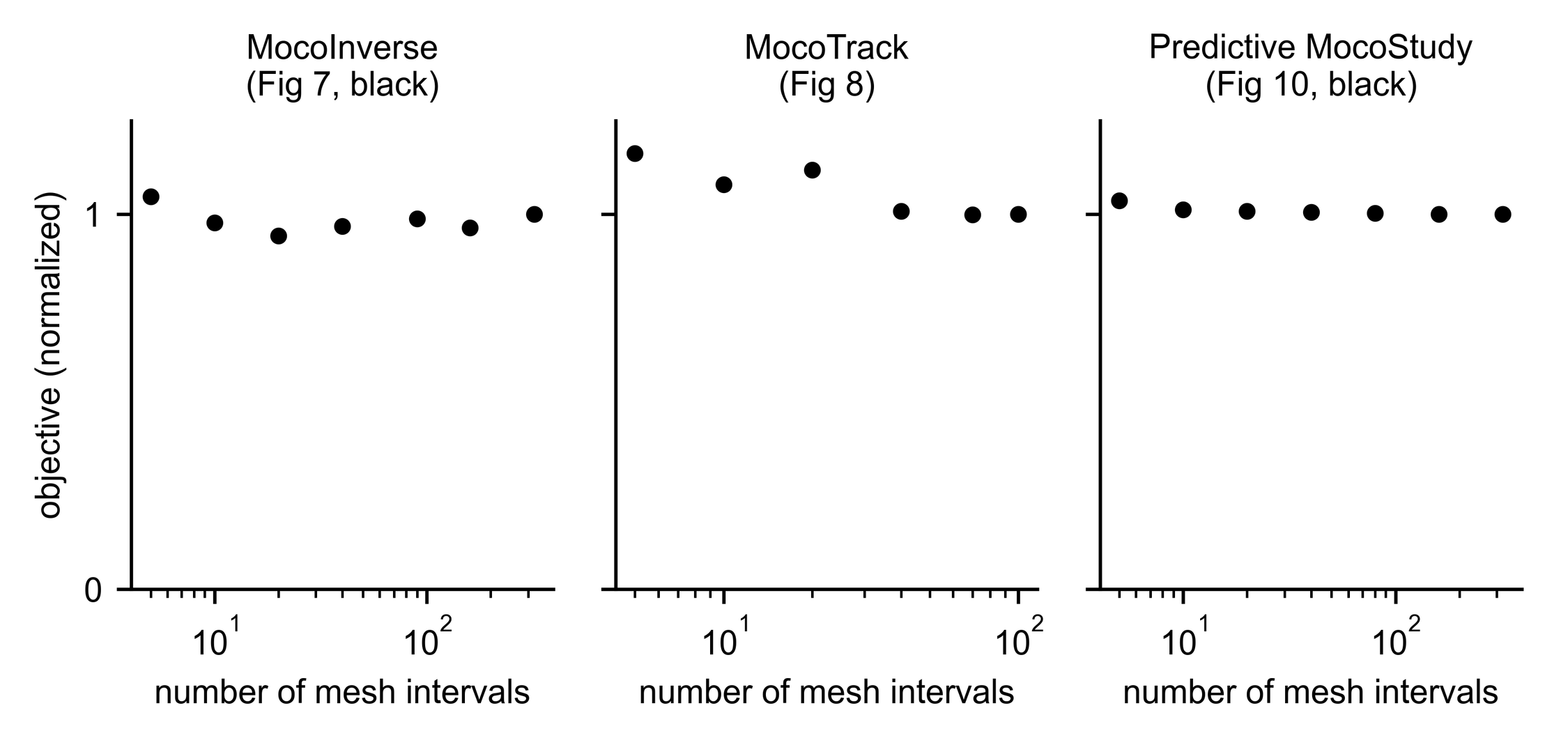

Supplement: S2 Fig — To ensure our results were not sensitive to the chosen number of mesh intervals (which is related to mesh density), we performed a convergence analysis: we ensured that the objective function value converged on a single value as the number of mesh intervals increased. We performed the convergence analysis for three problems from the results: “MocoInverse, gait, normal” (Fig 7, black), “MocoTrack, gait, normal” (Fig 8), and “Predictive MocoStudy, squat-to-stand, unassisted” (Fig 10, black). For each graph, the objective is normalized by the value of the objective from using the greatest number of mesh intervals. (TIF) [file pcbi.1008493.s003.tif]
